# Machine Learning and Network Analysis of Molecular Dynamics Trajectories Reveal Two Chains of Red/Ox-specific Residue Interactions in Human Protein Disulfide Isomerase

**DOI:** 10.1038/s41598-017-03966-5

**Published:** 2017-06-16

**Authors:** Razieh Karamzadeh, Mohammad Hossein Karimi-Jafari, Ali Sharifi-Zarchi, Hamidreza Chitsaz, Ghasem Hosseini Salekdeh, Ali Akbar Moosavi-Movahedi

**Affiliations:** 1 Department of Biophysics, Institute of Biochemistry and Biophysics, University of Tehran, Tehran, Iran; 2 Department of Molecular Systems Biology, Cell Science Research Center, Royan Institute for Stem Cell Biology and Technology, ACECR, Tehran, Iran; 3 Department of Bioinformatics, Institute of Biochemistry and Biophysics, University of Tehran, Tehran, Iran; 4 Department of Stem Cells and Developmental Biology, Cell Science Research Center, Royan Institute for Stem Cell Biology and Technology, ACECR, Tehran, Iran; 5 Computer Engineering Department, Sharif University of Technology, Tehran, Iran; 6 Computer Science Department, Colorado State University, Fort Collins, Colorado 80523 USA; 7 Department of Systems Biology, Agricultural Biotechnology Research Institute of Iran, Agricultural Research, Education, and Extension Organization, Karaj, Iran

## Abstract

The human protein disulfide isomerase (hPDI), is an essential four-domain multifunctional enzyme. As a result of disulfide shuffling in its terminal domains, hPDI exists in two oxidation states with different conformational preferences which are important for substrate binding and functional activities. Here, we address the redox-dependent conformational dynamics of hPDI through molecular dynamics (MD) simulations. Collective domain motions are identified by the principal component analysis of MD trajectories and redox-dependent opening-closing structure variations are highlighted on projected free energy landscapes. Then, important structural features that exhibit considerable differences in dynamics of redox states are extracted by statistical machine learning methods. Mapping the structural variations to time series of residue interaction networks also provides a holistic representation of the dynamical redox differences. With emphasizing on persistent long-lasting interactions, an approach is proposed that compiled these time series networks to a single dynamic residue interaction network (DRIN). Differential comparison of DRIN in oxidized and reduced states reveals chains of residue interactions that represent potential allosteric paths between catalytic and ligand binding sites of hPDI.

## Introduction

**Figure 1:**
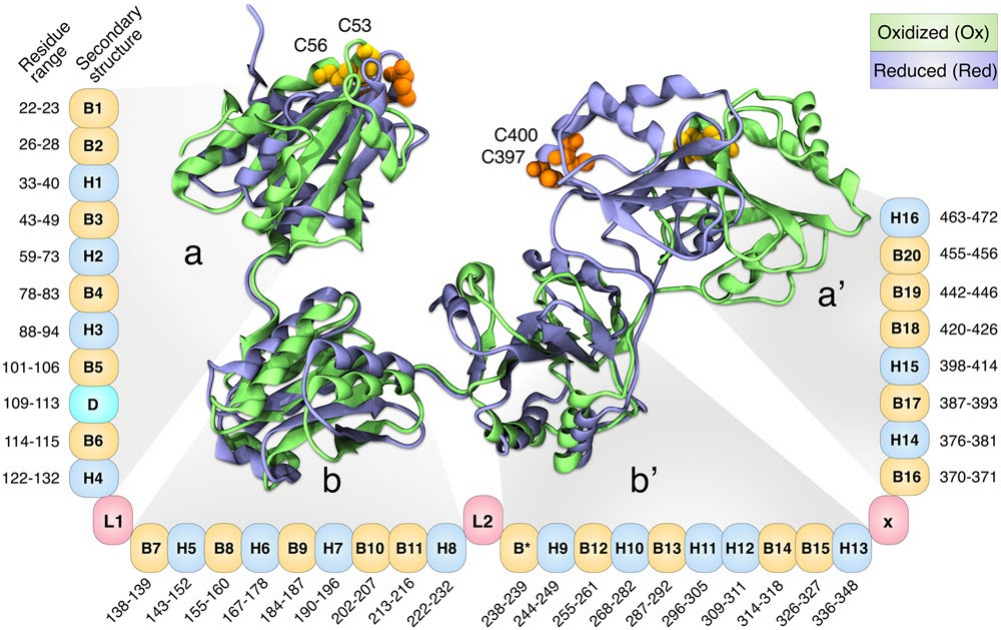
Structural representation of hPDI in oxidized and reduced states superimposed from bb’ domain along with secondary structure element of all residues. B: Sheet, H: Helix, D: Disordered region, L: Linker, X: X-linker. B*: Exclusive sheet in oxidized form.

The human protein disulfide isomerase (hPDI), is one of the most abundant redox-regulated molecular chaperones accounting for the folding of almost one-third of proteins in cells^1^. As the first discovered protein-folding catalyst^2^, hPDI works as both an enzyme and a chaperone in various cellular processes including the oxidative stress, unfolded protein response, apoptosis and viral membrane fusion, using thiol disulfide exchange reactions^3–6^. A wide range of multifunctional features of hPDI are tightly associated with its unique molecular architecture. The horseshoe-like structure of hPDI is composed of four thioredoxin-like domains named a, b, b’ and a’ (Fig. 1). The N- and C-terminal domains – a and a’– contain conserved cysteine residues within CGHC motifs that are responsible for the formation, breakage and rearrangement of disulfide bonds on peptide/protein substrates; whereas the b and b’ domains mostly contribute to substrate binding^7^. Targeted domain rearrangements during the redox-dependent activities of hPDI lead to the formation of two distinct “opened” and “closed” conformations in the oxidized (ox-hPDI) and reduced (red-hPDI) states, respectively^7–10^. Conformational transition of red/ox-hPDI is under the influence of a disulfide bond in the a’ domain, which leads to the rearrangements of b’ and a’ domains, followed by switching its enzymatic activities^7–11^. Despite the available crystal structures of both states^7^, different functions of these conformations still conceal behind their dynamical complexities. Some studies have investigated the relations among the inter-domain flexibility and its effects on global domain motions of partial and full human and yeast PDI using limited proteolysis, ﻿SAXS﻿, intrinsic fluorescence and NMR spectroscopies^9, 11–15^. However, limited differences have been reported between these two redox states in terms of their distinct dynamical functions. From this perspective, identification of the structural determinants that control the dynamical behavior of each state is crucial for better understanding of their functions.

Differences in the dynamical behaviors of ox- and red-hPDI are the results of complex geometrical and physicochemical interplays of many residues as the structural units. An important question is which residues play more critical roles and through what mechanisms. In recent years, conversion of the 3D structure of proteins into a 2D network of interacting residues has found useful applications in dealing with the complexity of biomolecular structures^16^. Some tools such as RINerator and RING have been utilized to construct residue interaction networks (RIN) by considering geometrical, physicochemical, evolutionary and energetics of each residue^17, 18^. Such networks could be used to compare and find hotspot players within the protein structure. However, the application of RIN is limited to the analysis of a single static snapshot of the protein structure obtained from experimental or computational resources. On the other hand, MD simulations are capable of producing a large number of conformations resulting from the time evolution of a protein. In these cases, each snapshot of the MD trajectory could be mapped to a corresponding RIN, and the dynamical analysis of such a huge number of networks would be a challenging task.

In the present work, we used MD simulations to reproduce the dynamical behaviors of hPDI in its oxidized and reduced states, independently. Regularities in large-scale domain motions of protein were then obtained through principal component analysis. Over the ensemble of the generated configurations, statistical machine learning methods were conducted to extract structural features that are related to the redox-dependent dynamics of proteins. By converting the dynamically sampled configurations to a sequence of residue interaction graphs, an approach was suggested, which provided a network based analysis of the dynamical differences of residue interactions between the two oxidation states.

## Results and Discussion

### Pattern of domain motions

**Figure 2:**
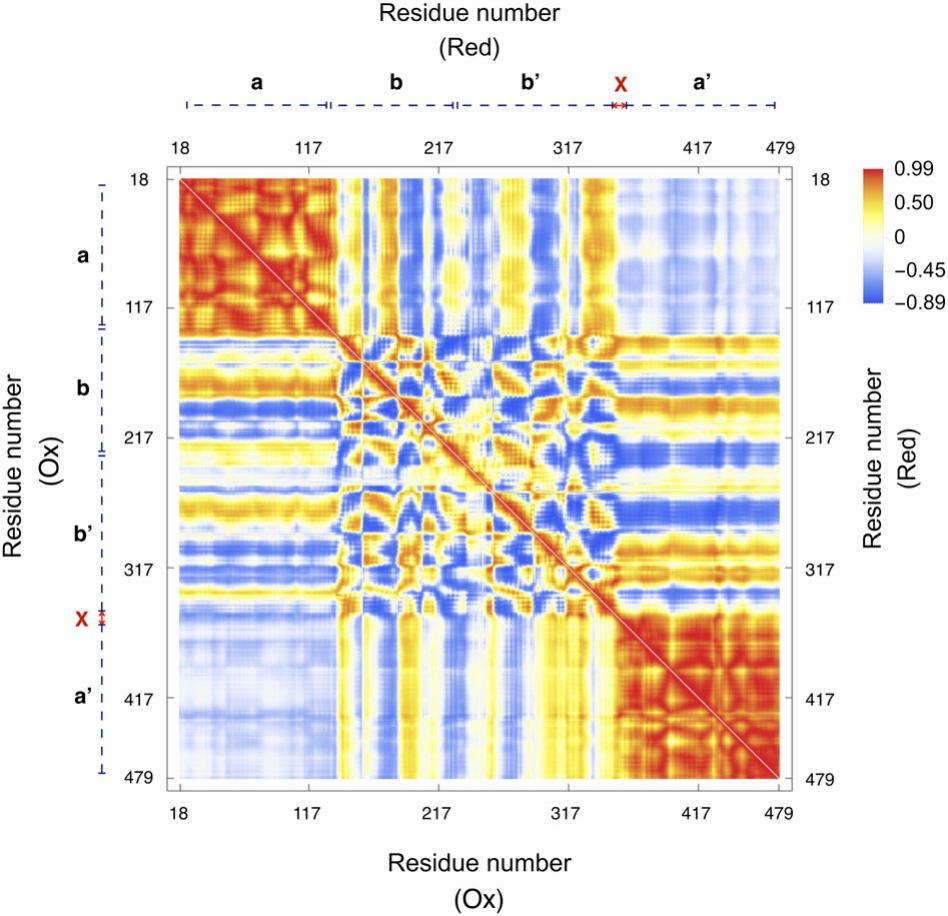
Cross-correlation matrix of atomic displacements around their average values for ox-hPDI (lower triangles) and red-hPDI (upper triangles). Values are ranged between 1 (red) and -1 (blue) for correlated and anti-correlated motions, respectively.

Time series of backbone (root mean square deviation) RMSD values were monitored with respect to the initial structures to check whether the recorded atomic movements were caused by the collective domain motions or irregular random ones. Some trajectories showed RMSD values as large as 15 Å for whole protein backbone while the single domain values were mostly less than 2 Å (see Figure S1). Accordingly, most of the captured structural variations could be assigned to large scale domain motions and each of the single domains exhibited considerable structural stability within its domain boundaries. Moreover, starting from multiple initial configurations provides a more efficient sampling of the conformational space of protein. Collective atomic motions were characterized by cross-correlation matrix of atomic displacements around their average values (see Fig. 2). Clear domain boundaries and blocked structure of correlations were in agreement with patterns of RMSD values. Since all trajectories were superimposed to bb’ domain, the central bb’ blocks in Fig. 2 mainly reflect the intra-domain correlations, while other blocks reflect the inter-domain collective trends of atomic motions. The same overall patterns of correlations were obtained for both the oxidized and reduced trajectories. In both systems, terminal domains showed anti-correlated motions with respect to each other which is necessary for opening-closing rearrangements.

**Figure 3:**
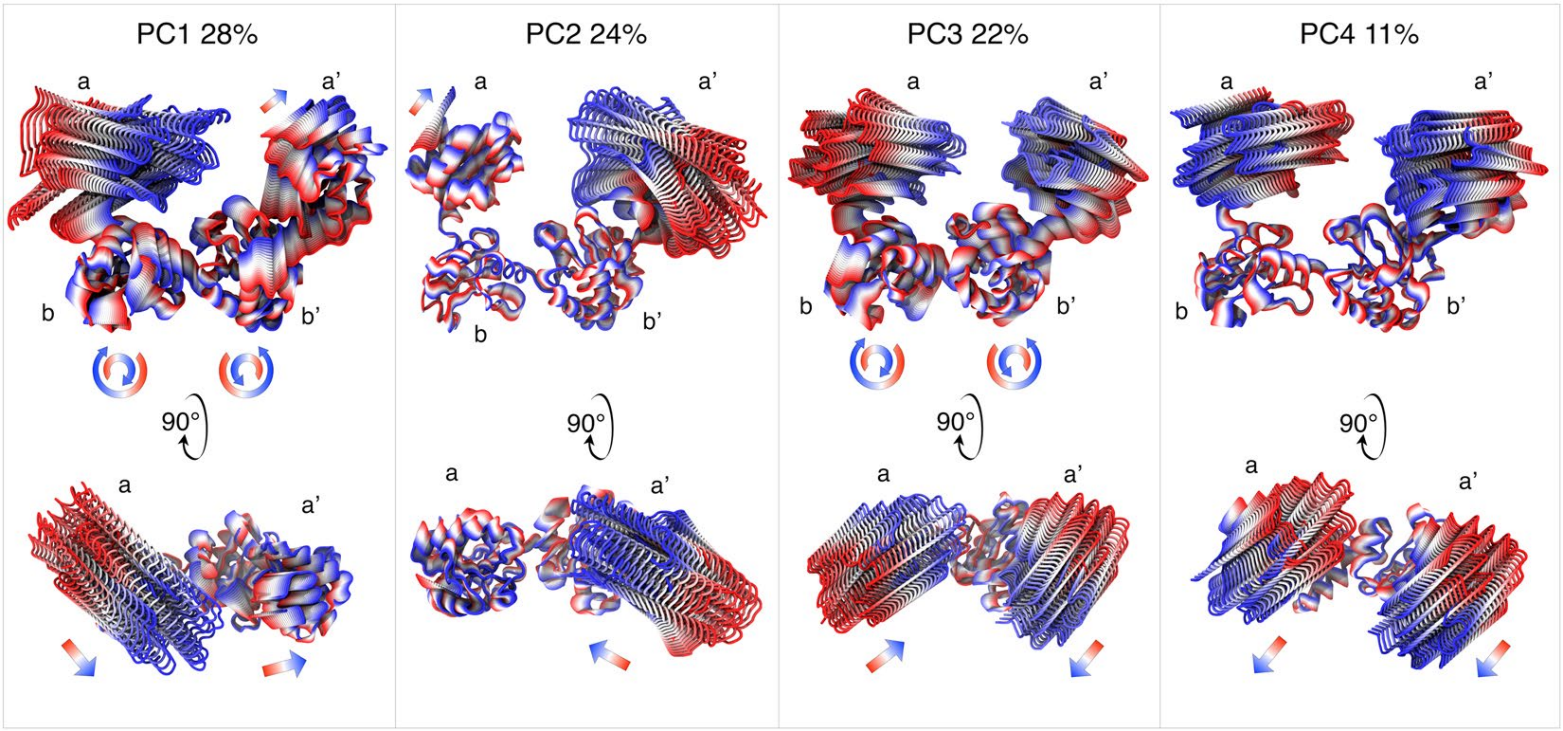
hPDI domain motion modes from PC1, PC2, PC3 and PC4 represented the dominant motions.

Essential modes of domain motions in hPDI were obtained from principal component analysis (PCA). The cumulative contribution of principal components (PCs) in total variance is shown in Figure S2. The first four PCs cover around 85% of total variance. As can be seen in Fig. 3, these essential components were different forms of hinge motions of domains a and a’ with respect to the bb’ base of protein. Single-domain hinge bending of terminal domains was the dominant motion in PC1 and PC2 while central domains showed a limited structural variation. In PC3 and PC4, both a and a’ were involved in a concerted hinge motion with antiparallel and parallel directions, respectively. The antiparallel bending has a dominated contribution in total variance in agreement with the patterns in cross-correlation matrix as described previously. Moreover, PC3 imitated movements of the a and a’ domains towards each other which could support previous studies showing the possibility of electron transfer between a and a’ domains during the catalytic activities^13, 19^. The extent of the large amplitude domain motions identified in the current study was in agreement with a recent computational analysis of yeast PDI^12^. They reported a range of 15–80 Å for sampled values of inter-site distances. Nearly the same range of values was obtained here (Figure S3). The physical plausibility of fully closed conformations will be discussed in the next section.

It should be noted that while the hinge motions of a and a’ domains were more highlighted in the first four PCs, there was a minor clockwise and counterclockwise twisting motion in b and b’ domains which increased the amplitude of movements in the terminal domains. A quite different form of collective motion could be found in PC5 and PC6, which was a twisting of terminal domains around ab and a’b’ inter-domain axes, respectively (Figure S4). Although a small fraction of structural variance was covered by these rotational movements, they are important for correct arrangement of catalytic sites towards substrates. The PCA approach was also used to extract basic modes of motion in two-domain parts of protein including ab, bb’ and b’a’ (Figure S5). For all domain pairs, similar types of motion were obtained including two bending hinge-type motions in orthogonal directions and one twisting rotation along inter domain axis. Complicated four-domain motions were thus deduced from similar two-domain movements.

### Redox-dependent opening-closing routes

**Figure 4:**
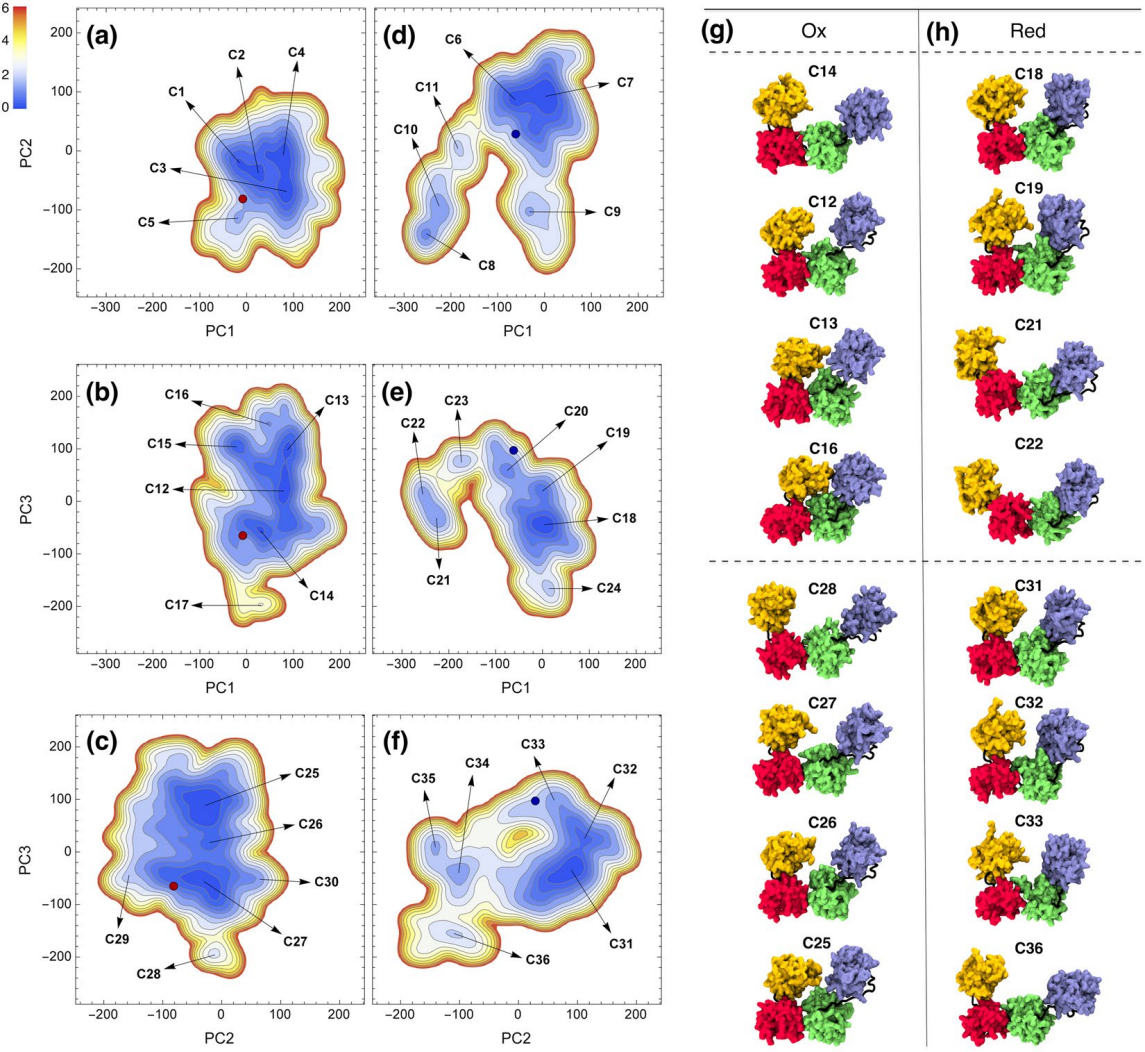
Projections of free energy landscape on two-dimensional spaces spanned by PC1-PC2, PC1-PC3 and PC2-PC3 in oxidized (**a**,**b** and **c**) and reduced (**d**,**e** and **f**) conformations. Conformers are numbered from C1 to C36. Representative conformers of FEL minima in oxidized (**g**) and reduced (**h**) states of hPDI. Yellow, red, green and blue stand for a, b, b’ and a’ domains, respectively.

Projections of free energy landscape (FEL) on two-dimensional spaces spanned by PC1-PC2, PC1-PC3 and PC2-PC3 were plotted in Fig. 4a–f. Some of the representative conformations presented in these figures, were depicted in Fig. 4g and h. The whole set of conformers could be found in Figures S6 and S7. Structural features of these conformers were reported in Table S1. All FEL projections of ox- and red-hPDI showed well-separated minima for open and closed conformations connected with low barriers. As could be seen in Fig. 4g, a path starting from open conformation of oxidized X-ray structure, passes through C14 and C12 conformations to reach C13 and C16 which were closed conformations. A similar closing route could be identified for ox-hPDI in PC2-PC3 subspace starting from C28, passing through C27 and C26 and reaching the C25 conformation. Comparison of these paths on FEL revealed that the ox-hPDI can easily switch between open and close states. In contrast, red-hPDI seems to be more restricted to intermediated semi-closed conformations similar to its X-ray structure. These semi-closed states were represented by a wide basin including C18, C19 and C20 conformations in PC1-PC3 subspace, or C31, C32 and C33 in PC2-PC3 subspace. In all of these states, the cleft between domains a and a’ was not completely closed but was not as open as C34 and C21 conformations. Open states like C34, C35 and C36 in PC2-PC3 subspace or C21, C22 and C23 in PC1-PC3 subspace, were sampled in red-hPDI trajectories but FEL plots showed that they were less stable and less accessible from semi-closed states due to higher barriers. Moreover, fully closed conformations with a completely diminished cleft between a and a’ domains were just seen on FEL of ox-hPDI (see C13, C16 and C25 conformations and their locations on FEL in Fig. 4). These opening-closing routs play a crucial role in regulation of substrate binding to hydrophobic pocket as reported in recent study about binding of α-synuclein to hPDI^20^.

### Redox-dependency in dynamics of structural features

To uncover which structural features of the hPDI could better discriminate between ox and red states, we employed both linear and non-linear classification methods^21^. With application of linear classifiers, we aimed to determine whether some structural features (e.g. inter-domain distances, angles, torsion or inter-residue distances) could discriminate ox and red snapshots of MD simulation by a simple threshold. We also used Support Vector Machine (SVM) with radial-basis kernel as a widely-used non-linear classifier since it could highlight more-complicated discriminative patterns in structural features such as bimodal distributions of domain level features (see Methods for more details).

**Figure 5:**
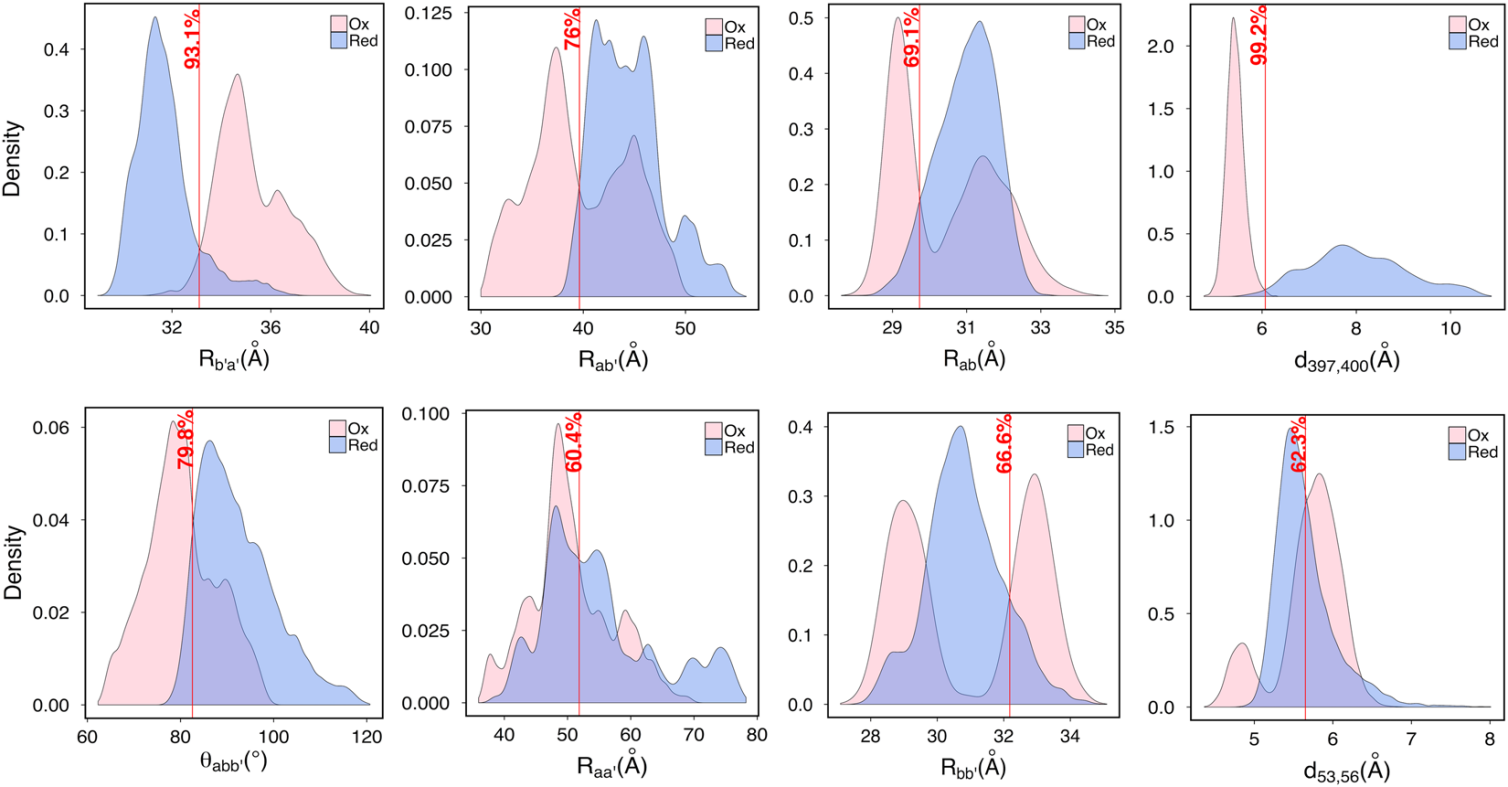
Domain and residue level distance distributions and their discrimination accuracies between the oxidized and reduced states of hPDI. Vertical red lines represent discrimination accuracy percentage. R: Domain distance; d: Residue distance; ${\rm{\theta }}$: Inter-domain angle.

Accuracy of linear and non-linear classifiers in discrimination of red/ox states, based on different domain level features, were reported in Table 1. As can be seen, the maximum discrimination was obtained from the inter-domain distance R_b’a’_ with an accuracy of 93%. The next most discriminative features were the inter-domain angle ѳ_abb’_ and inter-domain distance R_ab’_ with classification accuracies of 80% and 76%, respectively. Different red/ox behavior of R_b’a’_ was also reflected in its separated distribution of values over ox- and red-hPDI trajectories in Fig. 5. Distributions of ѳ_abb’_ and R_ab’_ had a considerable Red/Ox overlap but showed that the a and b’ domains could be in closer contact with each other in ox-hPDI. Fully closed conformations as identified on ox-hPDI FEL were thus the result of the elongated distance between b’ and a’ domains, which permits domain a to be in close contact with b’. The wide range of sampled R_b’a’_ values in both ox- and red-hPDI was consistent with previous works that report the X-linker as the most flexible region of the protein^9^. However, it seems that the oxidized state was affected by the flexibility of the X-linker, to a higher extent. The underlying mechanism resulting in different values of R_b’a’_ in ox- and red-hPDI systems is discussed below. X-ray structures suggest different restricted values for R_aa’_ in ox- and red-hPDI but as could be seen in Fig. 5, they sampled a wide range of inter-domain distances between 35 to 80 Å, which indicates that both states have the ability of synergic cooperation of catalytic and chaperone activities upon various substrates.

**Table 1:** Accuracy of non-linear (SVM) and linear discriminator in classification of red/ox states based on different domain level features.

Features	R_ab_	R_ab'_	R_aa'_	R_bb'_	R_ba'_	R_b’a'_	θ_abb'_	θ_bb’a'_	Φ_abb’a'_
Linear discriminator	69%	76%	60%	67%	72%	93%	80%	60%	69%
Non-linear discriminator (SVM)	73%	76%	62%	80%	72%	93%	80%	61%	75%

R: inter-domain distance; θ: angle; Φ: dihedral torsion.

**Figure 6:**
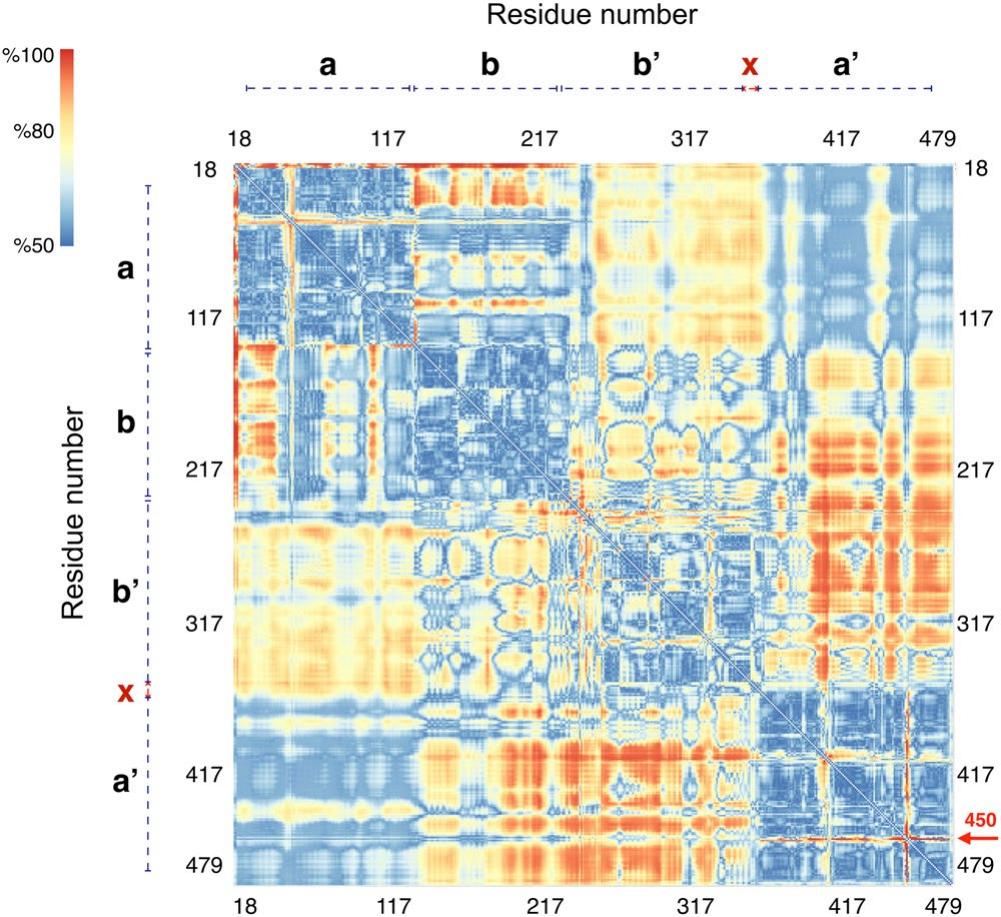
Pairwise residue discrimination matrix between oxidized and reduced state of hPDI. Values range from 50% (blue) to 100% (red) discrimination accuracies.

Accuracy of the linear method in the case of residue-residue distances *d*_*ij*_ was presented as a residue-residue discrimination accuracy matrix (Fig. 6). Each element of matrix represents the accuracy of red/ox classification of conformations based on their *d*_*ij*_ values. Four domain features of protein were reflected in this matrix as indicated by the appearance of different intra- and inter-domain blocks. The most discriminative inter-domain *d*_*ij*_ values, were located between b’ and a’ domains, which was an expected result due to the different behaviors of R_b’a’_ in ox- and red-hPDI. However, in the case of intra-domain blocks, there were some hotspot residues that exhibited quite different distances with other residues in the same domain between ox- and red-hPDI. Moreover, A450 and D18 had different red/ox behavior which was highlighted as distinct red lines in corner aa and a’a’ blocks, respectively (of Fig. 6). It should be noted that the A450 residue is in the middle of a loop connecting B19 and B20 sheets (L_B19-B20_). This loop had a conserved ω-shaped structure over red-hPDI trajectories that became slightly distorted in ox-hPDI state.

Another important emerged from the linear classifier accuracies was the differential red/ox behavior of Cys-Cys distances in domains a and a’. Accuracy of the red/ox classifications based on *d*_53,56_ and *d*_397,400_ were 62% and 99%, respectively, as was evident from their distributions in Fig. 5. In the absence of the disulfide bond, C397-C400 distance took larger values in domain a’ of red-hPDI as compared to that of ox-hPDI, while C53-C56 distance in domain a, showed the same range of values in both ox- and red-hPDI systems. This result was in agreement with previous studies showing the importance of redox sensitivity of the a’ compared to the domain a^10^.

### A differential dynamic residue interaction network can highlight possible roadmap of redox-specific conformational changes

**Figure 7:**
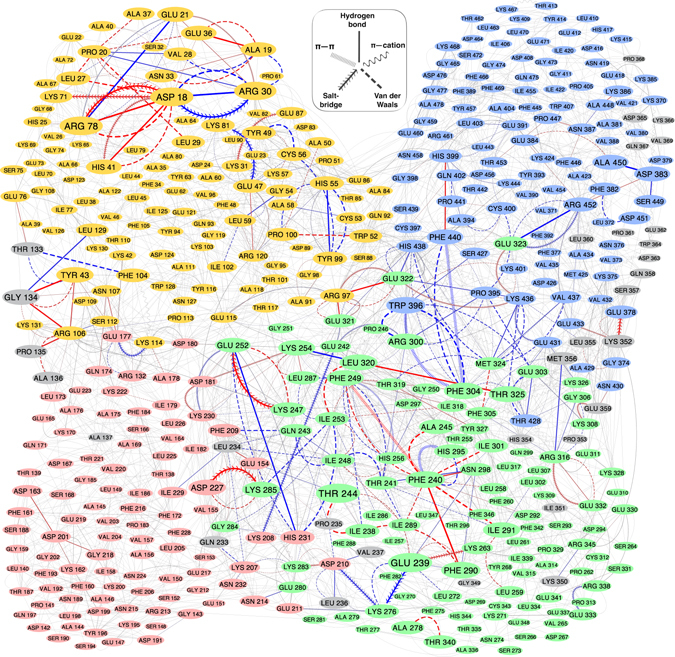
Dynamic residue interaction network (DRIN) in oxidized and reduced states of hPDI. The size of each vertex was assigned according to the maximum absolute weights of the edges arriving it. Sigmoid-like transformations from weights to the color or thickness of edges were used to show the interactions with unchanged weights between ox and red states as very thin grey edges, and the interactions with significantly higher persistence in one state as thick blue (red) or red (ox) edges. Nodes are colored by domains as yellow, red, green, blue and gray denote as domains a, b, b’, a’ and inter domain linkers, respectively.

hPDI conformational changes are influenced by the allosteric redox regulation of disulfide bonds. Both causality and energetics of the allosteric pathway identify the mechanisms governing the regulation of this action^22^. There are some methods that examine the causality of the perturbations within the allosteric path^23, 24^. Here, the time evolution of residue interaction networks were compiled to a dynamic residue interaction network named as DRIN. Since the DRIN graph reflected long lasting non-covalent interactions, a differential comparative DRIN graph could highlight central residues that are common or dominated in any of the ox- or red-hPDI systems. This representation was depicted in Fig. 7. As can be seen, the majority of red- or ox-dominated interactions occurred in a, b’ and a’ domains, which were responsible for substrate binding and enzymatic/chaperone activities of hPDI.

To test the importance of the identified hub residues of the DRIN graph on the functional activities of hPDI, we examined the situation of previously reported point mutations in differential DRIN graph (Table S2). Most of the point mutations that resulted in the alteration of hPDI activities were associated to the redox-dominated interacting residues. The majority of these mutations could affect the inter-domain coordination by changing the long-lasting interactions (Fig. 7 and Table S2). The network approach seems to be useful in identifying the critical residues in different oxidation states of hPDI.

The differential DRIN can explain the difference between ox- and red-hPDI in distribution of R_bb’_ by considering salt-bridges between D227, D210 and K208 in b domain with K285, K276 and E242 in the b’ domain, respectively. These interactions which were mainly red-dominated locked the R_bb’_ around its unimodal distribution of values while their absence in ox-hPDI permitted two domains to rest in two different distances as reflected by bimodal form of R_*bb*’_ distribution (see Fig. 5).

Another example of the redox-dependent adjustment of inter-domain linkers can be found in region D of domain a in Fig. 1. This part of the sequence showed a sheet-coil oscillation during MD trajectories (Figure S8). The oscillations seemed to be biased to the sheet in red-hPDI as a result of the hydrogen bond of R106 to S112, while in ox-hPDI, R106 mainly interacted with G134 and P135 in L1 linker.

### Discrete lockstitch-like interactions in oxidized and reduced hPDI

**Figure 8:**
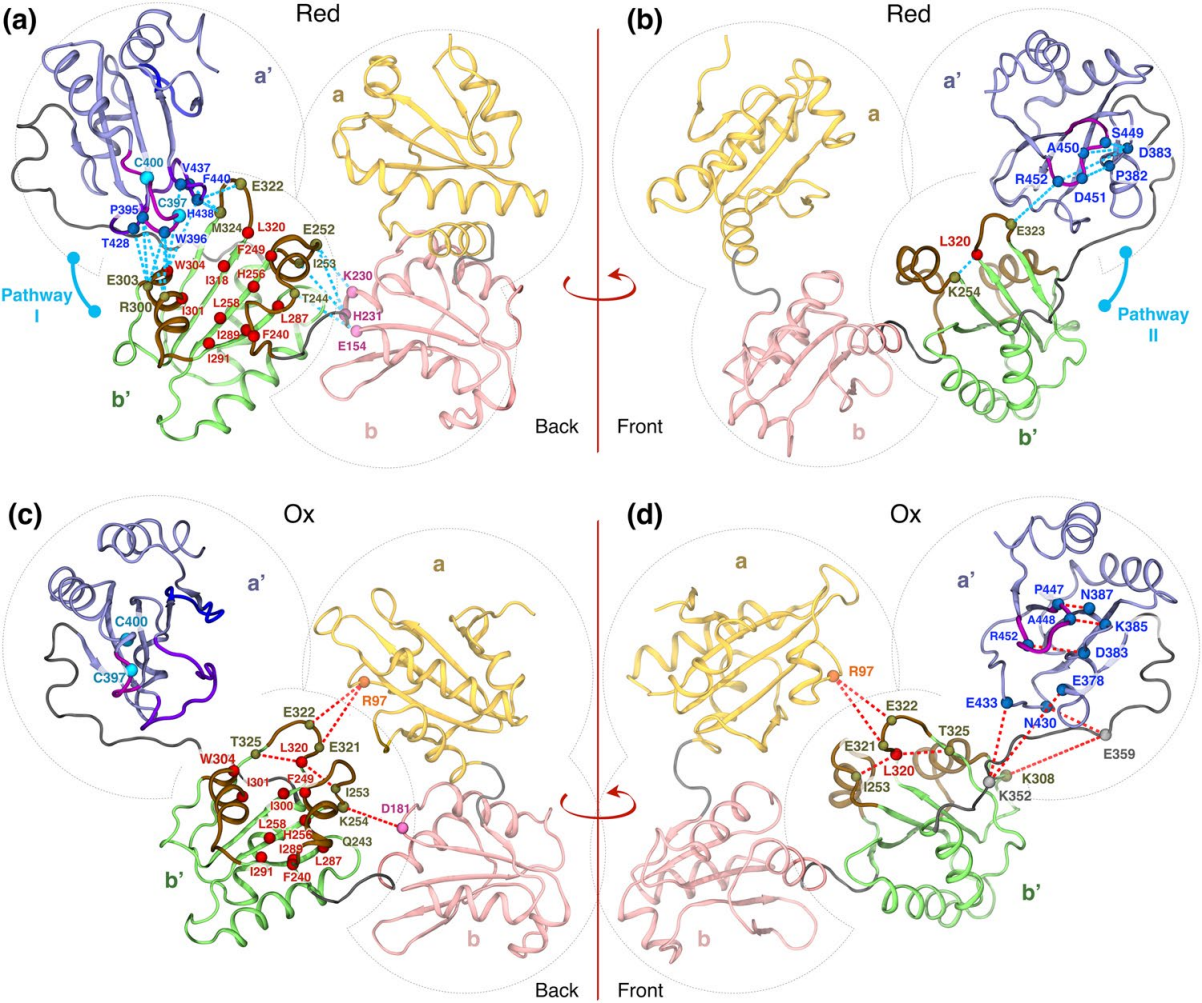
Red- (**a** and **b**) and ox- (**c** and **d**) dominated residue-residue interaction networks on two representative conformations of red- and ox-hPDI (C3 and C6 of FEL) between the b’ and a’ domains. Residues (C_α_) within the hydrophobic pocket in each state are colored in red. Red and blue dash-lines indicate the ox- and red-dominated interactions, respectively. Secondary structures involve in formation of ﻿the upper edge of﻿ hydrophobic ﻿pocket are colored in brown (H9, H11 and L_B14-B15_). Secondary structures of a’ domain that contribute in b’a’ interactions are colored in violet (L_B17-H15_, L_B18-B19_ and L_B19-B20_).

The machine learning approach used in this study revealed that b’a’ inter-domain distance is a critical feature of different conformational dynamics of hPDI in reduced and oxidized states. We therefore attempted to identify residue-level interactions that regulate this feature. In Fig. 8 important red- and ox-dominated pairwise residue interactions were depicted on two representative conformations of red- and ox-hPDI, respectively. Two sequential interaction pathways in red-hPDI held b’ and a’ domains close to each other. The first one, denoted as *pathway I*, links H11 (e.g. R300, E303 and F304) and L_B14-B15_ (e.g. E322 and M324) of the b’ domain to L_B17-H15_ (e.g. P395, W396) and L_B18-B19_ (e.g. K436, H438) of the a’ domain through hydrogen bonds, van der Waals and π-π stacking interactions (Fig. 8a and b). Therefore, the catalytic site of a’ (C397/C400) was connected to the core of hydrophobic pocket for further enzymatic/chaperone activities. The second sequence of interactions, denoted as *pathway II*, connected center of L_B14-B15_ (E323) in the b’ domain to L_B19-B20_ (R452) in the a’ domain via hydrogen bond and salt-bridge interactions (Fig. 8b). As noted before, these interactions keep the L_B19-B20_ loop in a conserved ω-shaped structure in red-hPDI. Two pathways (I and II) were connected to each other in the hydrophobic pocket of b’ domain by involvement of L_B14-B15_ (Fig. 8a and b). These interaction pathways act as a lockstitch that brings the b’ and a’ domains close to each other in red-hPDI.

In the absence of the red-dominated interaction of pathways I and II, different patterns of interactions occurred in ox-hPDI (Fig. 8c and d). The L_B19-B20_ loop in domain a’ became isolated with its own ox-dominated interactions, which distorted its ω-shaped structure. New interactions were also created between the a’ domain and the X-linker. In particular, interactions between K352 and E359 in X-linker with E378 and K308 in a’ seem to adjust the b’a’ distance indirectly based on the flexibility of X-linker (Fig. 8c and d).

Along with pathways I and II between the b’a’ domains, additional chains of interactions seem to adjust the size of hydrophobic pocket in oxidized and reduced conformations. As can be seen in Fig. 8a, the rim of hydrophobic pocket mainly regulated by two helices (H9 and H11) and a loop between the B14-B15 (L_B14-B15_). As mentioned earlier, residues within the H11 and L_B14-B15_ interacted with a’ domain in red-hPDI through pathway I. In addition, residues around H9 also form red-dominated interactions with the b domain (K230, H231 and E154) based on hydrogen bonds, salt-bridge and van der Waals interactions. On the other hand, the H9 position was controlled by the formation of ox-dominated interactions in the oxidized state (e.g. D181—K254). These results highlighted the importance of inter-domain long-lasting interactions in substrate selectivity of oxidized and reduced hPDI.

## Conclusion

hPDI plays diverse roles in various pathophysiological conditions such as the immune responses, coagulation, angiogenesis and even viral infection (reviewed in refs 25–27). Therefore, many attempts have been made to regulate its biological functions using small molecules for clinical applications^28^. Redox-associated structural changes are the key intrinsic feature to determine and control hPDI cross-functioning. For this propose, many studies have focused on finding the central residues that might play critical roles in manipulating different functions of hPDI^28, 29^. In this study, we aimed to identify the redox-specific structural features of hPDI at residue and domain levels.

hPDI intrinsically controls its functions by balancing the structural features with long-lasting non-covalent interactions. Differential dominated interactions within the redox-regulated conformations could explain some of the inter- and intra-domain cooperative arrangements and suggest the mechanisms that potentially preserve the oxidized and reduced conformations. In addition, comparative analysis of the differences between the identified dynamic residue interaction network, and the earlier experimental point mutations elucidate the relative structural impacts of redox-dominated interactions on the biological function of hPDI and propose critical locations for future allosteric small molecule design. This approach can also be used for the extraction and discrimination of subtle structural dynamic alterations and hub residues between the redox-dependent conformations of other redox-dependent enzymes.

## Methods

### Molecular Dynamics Simulation

X-ray crystal structures of ox-hPDI (PDB ID: 4EL1) and red-hPDI (PDB ID: 4EKZ) were used as the initial conformations for MD simulations^7^ using the CHARMM 27 force field in the NAMD 2.10 package^30^. Short missing regions were recovered using Modeller 9.13^31^. Missing hydrogens were added by Reduce 3.23^32^. Each system was then solvated in a rectangular box of TIP3P water molecules and neutralized by sodium ions. Both systems were minimized and carefully equilibrated via NPT dynamics at 300 K and 1 atm. A Nosé-Hoover Langevin piston and a Langevin thermostat were used to control the pressure and temperature^33, 34^, respectively. Periodic boundary conditions in conjunction with the particle mesh Ewald (PME) method were applied^35^. To accelerate the conformational sampling, an initial 10 ns trajectory was clustered based on backbone RMSD in the VMD 1.9 package^36^ and twelve representative structures (six for each system) from most populated clusters were used as starting points of 55 ns NPT dynamics. The whole procedure results in a sample of 156,000 structures from a total of 600 ns NPT simulation. More methodological details of MD simulations can be found in the supplemental text.

### Cross-Correlation and Principal Component Analysis

The backbone RMSD for each domain and whole protein was calculated along each trajectory with respect to the first production frame. The secondary structure characters of all residues were calculated over trajectories using the STRIDE program^37^. The overall rotation and translation of the protein were eliminated by alignment from Cα atoms of domains b and b’, since the bb’ pair of domains acts as a base for domain motions. To identify groups of residues with correlated motions, cross-correlation matrix of the atomic displacements was calculated^38^ (see SI text for details). To quantitatively address domain motions, some inter-domain geometric variables were calculated over trajectories. Six inter-domain distances denoted as *R*_*ij*_(*i*, *j* ∈ {*a*, *a*’, *b*, *b*’}), two inter-domain angles Θ_*abb*’_ and Θ_*bb*’*﻿a﻿*’_, and one torsion angle Φ_*abb*’*a*’_ were defined based on the domain centers (see Figure S9). Beside these mechanical “ball and spring” variable, a more comprehensive assessment of domain dynamics was performed via principal component analysis (PCA) of Cα Cartesian coordinates (see Supplementary data for details). The standard PCA was performed for the whole protein and two-domain subsets of coordinates including ab, bb’ and b’a’ domain pairs to obtain basic two-domain motions that results in complicated four-domain dynamics. The most important collective modes of motions were selected based on their contribution in total variance. The kernel density estimation with a Gaussian kernel was used for the construction of the probability distribution functions (PDFs) in subspaces spanned by pairs of principal components. These PDFs were then used for the construction of projected FEL.

To check the performance of adopted multi-trajectory approach in sampling of conformational space of hPDI, the inner products between PCA eigenvectors obtained from all data and those of different halves of the data were compared in Figure S10. Comparison of diagonal and off diagonal elements in panel b of Figure S10 shows that the directions of important collective motions would be the same if one considers only the half-length of all trajectories while this was not true for full-length of all trajectories. Accordingly, starting from multiple configurations was crucial for efficient sampling and for avoiding from trapping regions of landscape.

### Statistical Machine Learning Methods

Statistical machine learning methods were used to extract those structural features of protein which have a higher responsibility for the different dynamical behaviors of ox- and red-hPDI. Two types of structural variables were considered: *i*) Domain level features including *R*_*ij*_, Θ_*abb*_, Θ_*bb*’*a*’_ and Φ_*abb*’*a*_ *,* *ii*) Residue level features including pairwise residue-residue distances *d*_*ij*_, defined as the Euclidean distance between Cα atoms of residues *i* and *j* (see Figure S9). Values of the structural features in all trajectory snapshots were labeled as “ox” or “red” and a linear discrimination method was used for both residue and domain level features. In this regard, let *V* be any of the considered dynamical features, with observed ranges of values $[{V}_{min}^{ox},{V}_{min}^{ox}]$ and $[{V}_{min}^{{\rm{r}}{\rm{e}}d},{V}_{max}^{{\rm{r}}{\rm{e}}d}]$ in ox- and red-hPDI systems, respectively. The goal of the machine learning was to predict the “ox” or “red” label of each structure based on its *V* value. The linear method tried to find an optimum value denoted as *V*_*sep*_ from statistical distributions of *V* values in ox and red states. The features resulting in higher classification accuracy were assumed to be more important in description of dynamical differences between ox- and red-hPDI systems. Due to the bimodal distribution of some structural features (e.g. R_bb’_ distribution in ox states, see Fig. 5) we hypothesized that non-linear models might provide better classification accuracies than linear models, at least for a subset of features. To test this hypothesis, for each of 9 domain-level structural features (Table 1) we trained a separate Support Vector Machine (SVM) model to classify between ox and red states. The “svm” function of the “e1071” R package was used for this purpose, with default parameters and radial-basis kernel. To prevent overfitting, we assigned 156,000 available snapshots of both ox (N = 78,000) and red (N = 78,000) states into two disjoint training and test sets. Each snapshot was randomly assigned by equal probability to either training or test set, which resulted the sets to be of almost equal size. Each SVM model was trained based on one of the structural features to classify ox from red snapshots using training data, and then its performance was measured by test data. We observed identical classification accuracies between SVM and linear model for four structural features (R_ab’_, R_ba’_, R_b’a’_ and θ_abb’_) and higher SVM accuracies for five other features (R_ab_, R_aa’_, R_bb’_, θ_bb’a’_ and Φ_abb’a’_), which confirmed our hypothesis and also depicted that SVM models did not overfit to the training data (Table 1). As expected, R_bb’_ had the highest elevated accuracy between SVM and linear models (80% vs. 67%), which was due to the bimodal distribution of R_bb’_ in ox conformations (Fig. 5).

### Dynamic Residue Interaction Network

The standalone version of RING 2.0 program was used for calculation of residue interaction network (RIN)^17^. Five types of non-covalent interactions, including hydrogen bond, van der Waals, salt bridge, π-π and cation-π interactions were considered. Each of the 156,000 structures was converted to a graph with residues as its vertices and the pairwise interactions of the residues as its edges. There could be different edges between a pair of residues due to different types of non-covalent interactions. For each type of interaction, a separate adjacency matrix of a RIN graph denoted as *A*_*ij*_(*t*) was considered. From time series of *A*_*ij*_(*t*), the maximum interaction life time, ${{\rm{\Gamma }}}_{{ij}}$, was then calculated according to equation (1) to build the DRIN matrix denoted as ${\rm{\Gamma }}$ for each type of non-covalent interactions.

1 $${{\rm{\Gamma }}}_{ij}={\rm{\arg }}\,{{\rm{\max }}}_{\tau }\{\exists {\tau }_{0}:(\underset{t={\tau }_{0}}{\overset{\tau +{\tau }_{0}-1}{{\rm{\Pi }}}}{A}_{ij}(t))=1\}$$

By this definition, for each type of interaction, 156,000 RIN graphs were compiled to two single DRIN graphs for each of the ox- and red-hPDI systems. To highlight the differences between the oxidized and reduced states, we computed the differential DRIN graph ${\rm{\Delta }}$ with adjusted weights that represented the fold change of Γ_*ij*_ values between ox- and red-hPDI systems. The elements of the differential DRIN matrix Δ were computed for each type of interaction as equation (2):

2 $${{\rm{\Delta }}}_{ij}={{\rm{l}}{\rm{o}}{\rm{g}}}_{2}(\frac{{{\rm{\Gamma }}}_{ij}^{ox}+\varepsilon }{{{\rm{\Gamma }}}_{ij}^{{\rm{r}}{\rm{e}}d}+\varepsilon })$$

where *ε* is a small positive value, which is considered to prevent the division by zero. By considering a cutoff on the absolute values of Δ_*ij*_, we could identify the pairs of residues that had a significant alternated pattern of interactions between the ox and red states. For visualization and analysis purposes Cytoscape 3.3.0^39^ was used and differential DRIN graphs obtained for different types of interactions were merged together in a single graph with different edge styles.

## Electronic supplementary material

Supplementary_Info
