# Supplementary material for: Machine Learning and Network Analysis of Molecular Dynamics Trajectories Reveal Two Chains of Red/Ox-specific Residue Interactions in Human Protein Disulfide Isomerase
Source: Sci Rep. 2017 Jun 16;7:3666. doi: 10.1038/s41598-017-03966-5 (PMC5473932; doi:10.1038/s41598-017-03966-5)
Supplement: Supplementary file 1 — Supplementary_Info [file 41598_2017_3966_MOESM1_ESM.pdf]

TITLE

Machine Learning and Network Analysis of Molecular Dynamics Trajectories Reveal Two Chains of Red/Ox-specific Residue Interactions in Human Protein Disulfide Isomerase

Razieh Karamzadeh[1,2], Mohammad Hossein Karimi-Jafari[3*], Ali Sharifi-Zarchi[4,5,6], Hamidreza Chitsaz[6], Ghasem Hosseini Salekdeh[2,7], Ali Akbar Moosavi-Movahedi[1*]

[1] Department of Biophysics, Institute of Biochemistry and Biophysics, University of Tehran, Tehran, Iran.

[2] Department of Molecular Systems Biology, Cell Science Research Center, Royan Institute for Stem Cell Biology and Technology, ACECR, Tehran, Iran.

[3] Department of Bioinformatics, Institute of Biochemistry and Biophysics, University of Tehran, Tehran, Iran.

[4] Department of Stem Cells and Developmental Biology, Cell Science Research Center, Royan Institute for Stem Cell Biology and Technology, ACECR, Tehran, Iran.

[5] Computer Engineering Department, Sharif University of Technology, Tehran, Iran.

[6] Computer Science Department, Colorado State University, Fort Collins, Colorado 80523, USA.

[7] Department of Systems Biology, Agricultural Biotechnology Research Institute of Iran, Agricultural Research, Education, and Extension Organization, Karaj, Iran

* Corresponding authors

SUPPLEMENTARY INFORMATION

METHODS

Molecular Dynamic Simulations

X-ray crystal structures of ox-hPDI (PDB ID: 4EL1) and red-hPDI (PDB ID: 4EKZ) were used as initial conformations for MD simulations[1] with CHARMM 27 force field in the NAMD 2.10 package[2]. Short missing regions including residues 250 to 254, 321 to 323 and single residue 479 were recovered using Modeller 9.13[3]. Missing hydrogens were added by Reduce 3.23[4]. Each system was then solvated in a rectangular box of around 16491 TIP3P water molecules with at least 10 Å padding around protein and neutralized by addition of 23 Sodium ions. Both systems were minimized for 20000 steps of conjugate gradient method while harmonic restraints with a force constant of 10 kcal mol^{-1} $Å^{-2}$ were applied to protein heavy atoms. Gradual heat up from 10 to 300 K were then performed via 500 ps NPT dynamics with a time step of 1 fs while harmonic restraints were gradually relaxed. Nosé-Hoover Langevin piston was used to keep pressure at 1 atm and Langevin thermostat was used to control temperature[5,6]. Periodic boundary conditions were applied and non-bonded interactions were truncated at 12 Å using a smooth switching started at 10 Å. Long-range electrostatic interactions were calculated using the particle mesh Ewald (PME) method with a grid spacing of 1 Å[7]. After heat up and removal of restraints, the equilibration phase was continued at 300 K up to 2 ns. In subsequent MD simulations, the time step was increased to 2 fs and all bonds with hydrogen partners were kept rigid using the SHAKE algorithm. MD trajectories were recorded every 4 ps. To accelerate the conformational sampling, both systems were simulated for 10 ns and recorded structures were clustered based on their backbone RMSD via the quality threshold (QT) algorithm implemented in the VMD 1.9

package [8,9]. Six representative structures from most populated clusters of each of the ox-hPDI and red-hPDI systems were then used as starting points of 55 ns NPT dynamics and all recorded snapshots excluding those of the first 3 ns were used for subsequent analysis. This consists of 156000 structures from a total of 600 ns NPT simulation.

Cross Correlation and Principal Component Analysis

Backbone RMSD for each domain and whole protein was calculated along each trajectory with respect to the first production frame. Secondary structure character of all residues were calculated over trajectories using the STRIDE program[10]. To identify groups of residues with correlated motions, cross correlation matrix of atomic displacements was calculated according to equation (1):

$$c_{ij} = \langle(\vec{r}_i - \langle\vec{r}_i\rangle)(\vec{r}_j - \langle\vec{r}_j\rangle)\rangle \Big/ \sqrt{\langle(\vec{r}_i - \langle\vec{r}_i\rangle)^2\rangle\langle(\vec{r}_j - \langle\vec{r}_j\rangle)^2\rangle} \qquad \text{(Eq. 1)}$$

where c_{ij} is the cross correlation or normalized covariance between displacements of atoms i and j with position vectors $\vec{r}_i$ and $\vec{r}_j$, respectively [11]. The angular bracket $\langle\ \rangle$ represents time average over collected trajectory and Cα atoms were considered as reference for position of each residue. By this definition, c_{ij} elements, take values between -1 and 1. Extreme values -1 and 1 show average movements in opposite or the same direction, respectively, while a value near 0 corresponds to uncorrelated displacements or movement in orthogonal directions. Before calculation of cross correlation matrix, the overall rotation and translation of protein were eliminated from trajectory by alignment from Cα atoms of domains b and b' to the first frame of the first production trajectory. Though there are many arbitrary choices for such an alignment in a multi-domain protein, our choice is the most reasonable in the case of hPDI since the bb' pair of domains act as a base for domain motions.

To address domain motions in a more quantitative manner, some inter-domain geometric variables were defined and calculated over trajectories. Six inter-domain distances denoted as $R_{ij}\left(i, j \in \{a, a', b, b'\}\right)$ were defined between domain geometric centers. Two inter-domain angles $\Theta_{abb'}$ and $\Theta_{bb'a'}$ were defined between abb' and bb'a' domain centers, respectively. One torsion angle $\Phi_{abb'a'}$ was also defined as the angle between two intersecting planes formed by abb' and bb'a' domain centers. Beside these mechanical "ball and spring" variables, a more comprehensive assessment of domain dynamics was performed via principal component analysis (PCA) of Cα Cartesian coordinates. The standard PCA was performed by superimposition of trajectories on Cα atoms of domains b and b' and diagonalization of covariance matrix of atomic fluctuations to obtain corresponding eigenvectors and eigenvalues in descending order. MD trajectories were then transformed to the new space defined by eigenvectors. To compare oxidized and reduced systems, all trajectories were joined together for PCA. To analyze and visualize hPDI motions in reduced dimensions, the most important collective modes of motions were selected based on their eigenvalue magnitudes (i.e. their contribution in total variance). For visualization purposes principal components were transformed back to Cartesian coordinates. The same procedure was also repeated for two-domain subsets of coordinates including ab, bb' and b'a' domain pairs to obtain basic two-domain motions that results in complicated four-domain dynamics. The free energy landscape (FEL) spanned by the first two principal components PC1and PC2 is given by equation (2).

$$\Delta G(PC_1, PC_2) = -k_B T[\ln \rho(PC_1, PC_2) - \ln \rho_{\max}] \quad \text{(Eq. 2)}$$

where (PC1, PC2) is the probability distribution function obtained from MD data and $\rho_{\max}$ is its maximum value which is subtracted to put zero of free energy on the most probable

conformation. The kernel density estimation with a Gaussian kernel was used for construction of probability distribution functions. Projected FELs were also obtained for PC1-PC3 and PC2-PC3 subspaces.

To check the performance of adopted multi-trajectory approach in sampling of conformational space of hPDI, the inner products between PCA eigenvectors obtained from all data and those of different halves of the data were compared in Figure S10. Comparison of diagonal and off diagonal elements in panel b of Figure S10 shows that the directions of important collective motions would be the same if one considers only the half-length of all trajectories while this is not true for full-length of all trajectories. Accordingly, starting from multiple configurations is crucial for efficient sampling and for avoiding from trapping regions of landscape.

Statistical Machine Learning Methods

Two types of structural variables were considered to discriminate dynamical behavior of ox- and red-hPDI: *i*) Domain level features including R_{ij}, $\Theta_{abb'}$, $\Theta_{bb'a'}$ and $\Phi_{abb'a'}$. *ii*) Residue level features including pairwise residue-residue distances d_{ij}, defined as the Euclidean distance between Cα atoms of residues i and j. Values of these parameters were collected from all trajectory snapshots and labeled as "ox" or "red". To find those features that can discriminate oxidized states from reduced ones, a binary Support Vector Machine (SVM) was used to classify conformations as oxidized or reduced states. Those features that result in higher classification accuracy are assumed to be more important in description of dynamical differences between ox- and red-hPDI systems. Let V be any of the considered dynamical features, with observed ranges of values $\left[V_{\min}^{Ox}, V_{\max}^{Ox}\right]$ and $\left[V_{\min}^{\mathrm{Re}d}, V_{\max}^{\mathrm{Re}d}\right]$ in ox- and red-hPDI systems, respectively. The question is to what extent the red/ox state of the protein can be

determined solely based on V values. In a special case with $\left[V_{\min}^{Ox}, V_{\max}^{Ox}\right] = \left[V_{\min}^{\mathrm{Re}d}, V_{\max}^{\mathrm{Re}d}\right]$ the state of the protein cannot be determined accurately based on the observed V values. On the other hand, if $\left[V_{\min}^{Ox}, V_{\max}^{Ox}\right] \cap \left[V_{\min}^{\mathrm{Re}d}, V_{\max}^{\mathrm{Re}d}\right] = \phi$, there is no overlap between sampled ranges of V values in red and ox trajectories and a given frame from an unknown trajectory can be classified, at 100% accuracy, to either ox or red state, depending on its V value. For instance, if $V_{\max}^{Ox} < V_{\min}^{\mathrm{Re}d}$ then a threshold of V_{sep} in the open range $\left(V_{\max}^{Ox}, V_{\min}^{\mathrm{Re}d}\right)$ can be used to discriminate the red/ox state of any hPDI structure. In other cases with limited overlap between $\left[V_{\min}^{Ox}, V_{\max}^{Ox}\right]$ and $\left[V_{\min}^{\mathrm{Re}d}, V_{\max}^{\mathrm{Re}d}\right]$ ranges, we used a custom linear discriminator to find an optimum value for V_{sep} from statistical distributions of V values in ox and red states which maximizes the accuracy of ox vs. red discrimination based on the value V of a given snapshot. We also used the SVM with radial-basis kernel to improve ox vs. red discrimination accuracy over the linear models. Although this method could not provide a single threshold V_{sep}, it significantly improved the discrimination accuracy in several cases, particularly when the distribution of V was bimodal or multi-modal in some state. In all cases the classifier was trained with 50% random selection of all available frames (in both ox and red states) and tested with the rest of the frames. (Code available upon request)

Dynamic Residue Interaction Network (DRIN)

The standalone version of RING 2.0 program was used for calculation of residue interaction network (RIN). Five types of non-covalent interactions were considered including hydrogen bond, van der Waals, salt bridge, π-π and cation-π interactions. The RING was used in a mode that reports multiple interactions per residue pair but only one interaction per interaction type. Accordingly, each of the 156000 structures was converted to a graph with residues as its vertices and the pairwise interactions of the residues as its edges. There could

be different edges between a pair of residues due to different types of non-covalent interactions.

Let n be the number of residues and $A_{ij}(t)$ be the $n\times n$ adjacency matrix of a RIN graph for each type of non-covalent interaction. The $A_{ij}(t)$ elements are equal to zero or one for the absence or presence of considered type of interaction between residues i and j in time t of some MD trajectory. From time series of $A_{ij}(t)$, the maximum interaction life time, Γ_{ij}, was then extracted to build the matrix Γ which will be denoted here as the DRIN matrix for considered type of non-covalent interaction. Here we define Γ_{ij} as the maximum length of a continuous period of time in which a persistent interaction type is observed in the RIN graphs between the residues i and j:

$$\Gamma_{ij} = \arg\max_{\tau} \left\{ \exists \tau_0 : \left(\prod_{t=\tau_0}^{\tau+\tau_0-1} A_{ij}(t) \right) = 1 \right\} \qquad \text{(Eq. 3)}$$

By this definition, for each type of interaction, 156000 RIN graphs were compiled to two single DRIN graphs for each of the ox- and red-hPDI systems. The simpler choices, such as summing $A_{ij}(t)$ for each pair of residues during the whole period of time, results in noisy and transient on-and-off interactions often make a big total which could often dominate the effect of persistent interactions. Accordingly, a DRIN matrix can be considered as a complete $n\times n$ weighted graph with edge weights equal to Γ_{ij}. The DRIN matrices $\Gamma_{ij}^{\mathrm{Ox}}$ and $\Gamma_{ij}^{\mathrm{Red}}$ were calculated for each of the ox- and red-hPDI systems, respectively, and for all five types of non-covalent interactions considered. Values of Γ_{ij} elements were averaged over six separate trajectories of each system.

To highlight differences between oxidized and reduced states, we computed the differential DRIN graph Δ with the same nodes and edges as DRIN graph, but adjusted weights that represent the fold change of Γ_{ij} values between ox- and red-hPDI systems. More specifically, the elements of the differential DRIN matrix Δ were computed for each type of interaction as:

$$\Delta_{ij} = \log_2\left(\frac{\Gamma_{ij}^{Ox} + \varepsilon}{\Gamma_{ij}^{\mathrm{Re}\,d} + \varepsilon}\right) \qquad \text{(Eq. 4)}$$

In the above equation, ε is a small positive value with negligible effect on the outcome, which is considered to prevent the division by zero. In our study, we set ϵ equal to the time between two consecutive MD snapshots that means we have elongated the maximum duration of each interaction by 1 more frame. This is negligible with respect to the values of Γ_{ij} for persistent interactions that were in the order of hundreds or thousands of frames.

We used the differential DRIN matrix in a number of ways. By considering a cutoff on the absolute values of Δ_{ij} we could identify the pairs of residues that had a significant alternated pattern of interactions between ox and red states. We could also consider Δ as the weights of a graph, where positively and negatively weighted edges depict the interactions that are more persistent in ox and red states, respectively. We visualized such a network using the Cytoscape version 3.3.0, by assigning the colors and the thickness of the edges according to the interaction weight in differential DRIN matrix. For visualization and analysis purposes differential DRIN graphs obtained for different types of interactions were merged together in a single graph with different edge styles.

REFERENCES

1. Wang, C. C.-C. *et al.* Doncheva. *Antioxid. Redox Signal.* **19,** 36–45 (2013).

2. Phillips, J. C. *et al.* Scalable molecular dynamics with NAMD. *Journal of Computational Chemistry* **26,** 1781–1802 (2005).

3. Eswar, N., B. Webb, M.A. Marti-Renom, M.S. Madhusudhan, D. Eramian, M.-Y. Shen, U. Pieper, and A. Sali. 2007. Comparative protein structure modeling using MODELLER. Curr. Protoc. Protein Sci. 50:2.9:2.9.1–2.9.31.

4. Word, J. M., Lovell, S. C., Richardson, J. S. & Richardson, D. C. Asparagine and glutamine: using hydrogen atom contacts in the choice of side-chain amide orientation. *J. Mol. Biol.* **285,** 1735–1747 (1999).

5. Martyna, G. J., Tobias, D. J. & Klein, M. L. Constant pressure molecular dynamics algorithms. *J. Chem. Phys.* **101,** 4177 (1994).

6. Feller, S. E., Zhang, Y., Pastor, R. W. & Brooks, B. R. Constant pressure molecular dynamics simulation: The Langevin piston method. *J. Chem. Phys.* **103,** 4613 (1995).

7. Darden, T., York, D. & Pedersen, L. Particle mesh Ewald: An $N \cdot \log(N)$ method for Ewald sums in large systems. *J. Chem. Phys.* **98,** 10089–10092 (1993).

8. Humphrey, W., Dalke, A. & Schulten, K. VMD: Visual molecular dynamics. *J. Mol. Graph.* **14,** 33–38 (1996).

9. Heyer, L. J., Kruglyak, S. & Yooseph, S. Exploring expression data identification and analysis of coexpressed genes. *Genome Res.* **9,** 1106–1115 (1999).

10. Frishman, D. & Argos, P. Knowledge-based protein secondary structure assignment. *Proteins Struct. Funct. Genet.* **23,** 566–579 (1995).

11. Ichiye, T. & Karplus, M. Collective motions in proteins: a covariance analysis of

atomic fluctuations in molecular dynamics and normal mode simulations. *Proteins* **11,** 205–217 (1991).

SUPPLEMENTARY FIGURES

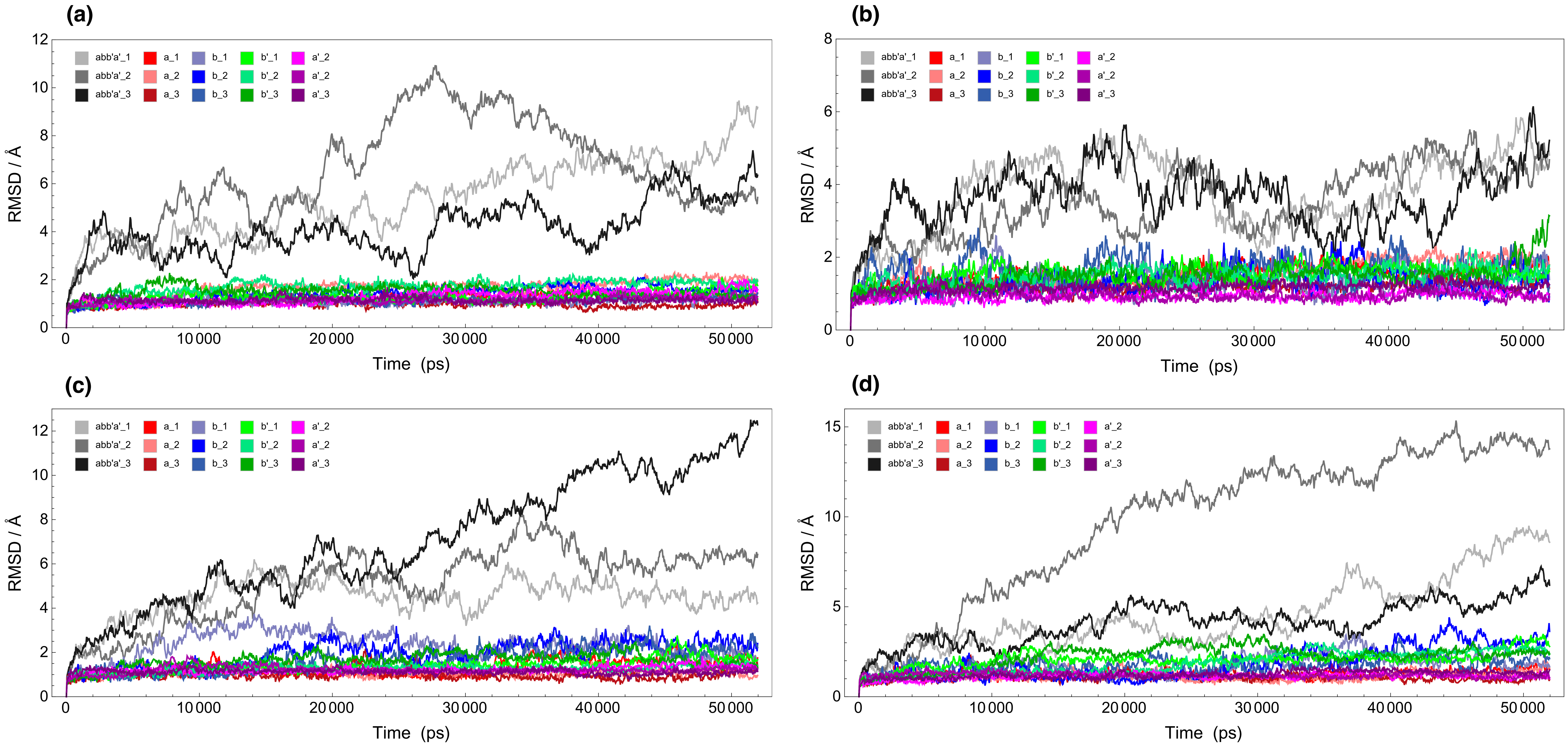

Figure S1 backbone RMSD values with respect to the initial structure for single domains and whole protein in oxidized (**a** and **b**, respectively) and reduced (**c** and **d**, respectively) hPDI conformations.

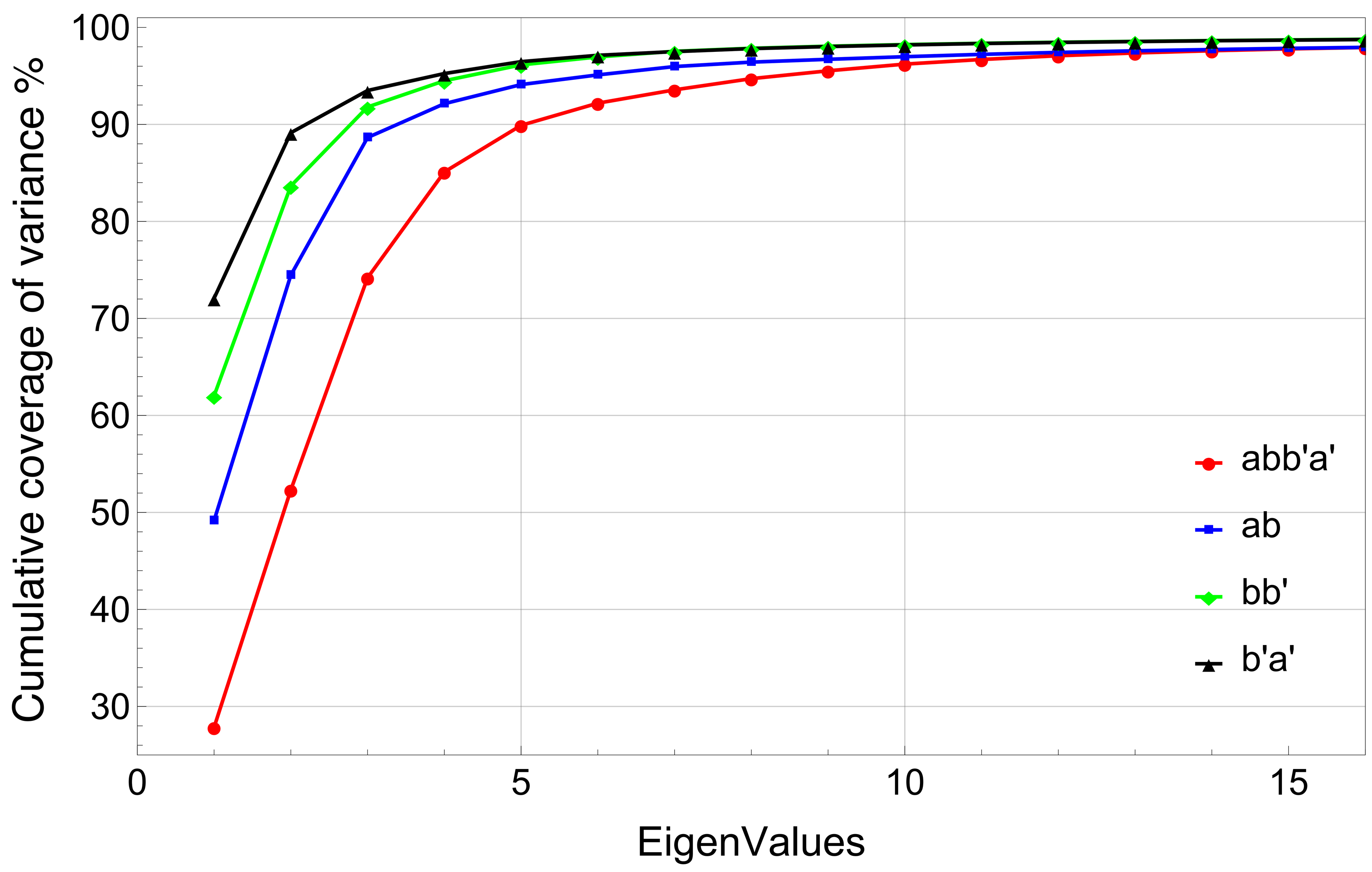

Figure S2 The cumulative contribution of principal components in total variance.

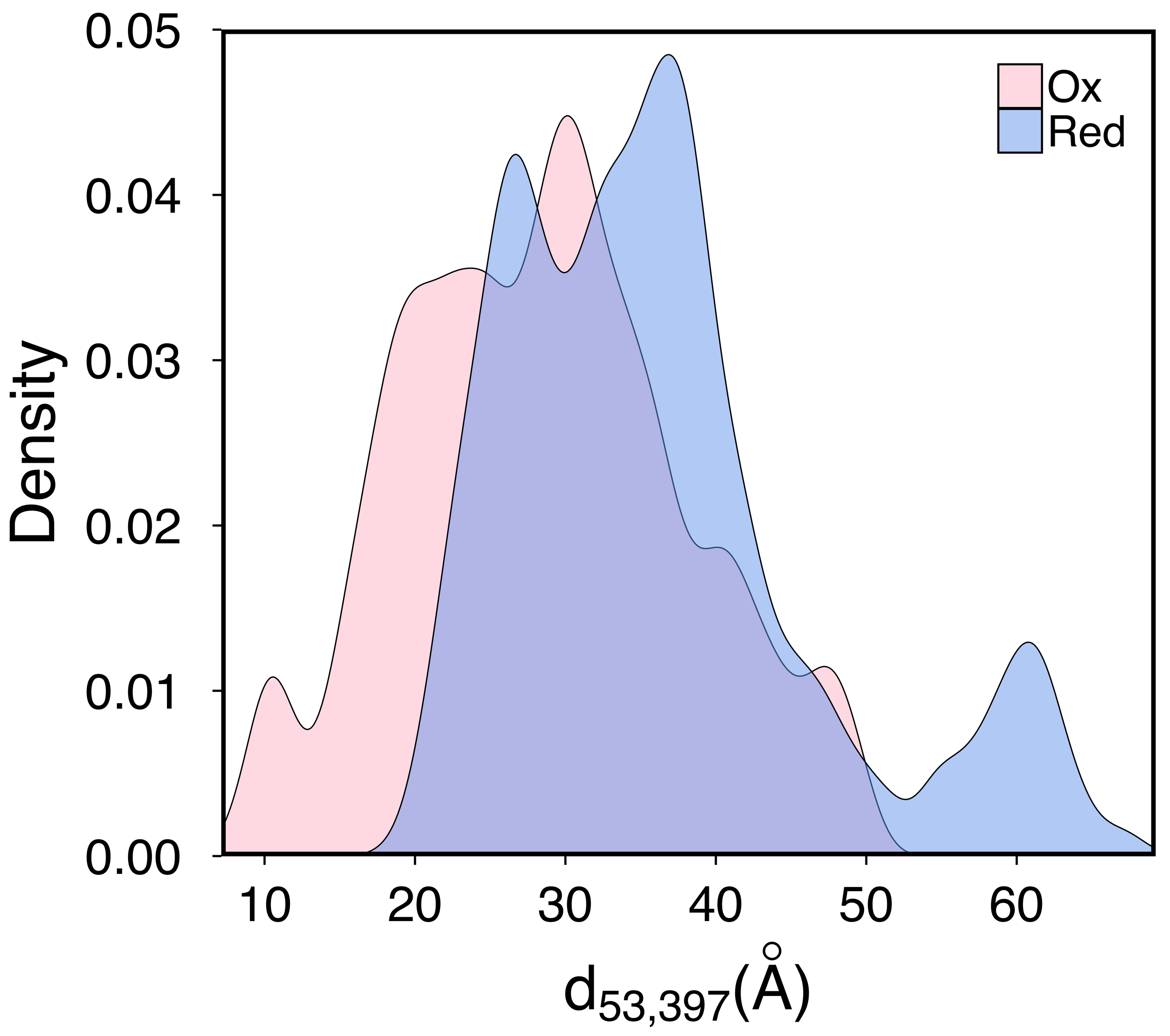

Figure S3 Distance between the catalytic Cysteines of the a and a' domains.

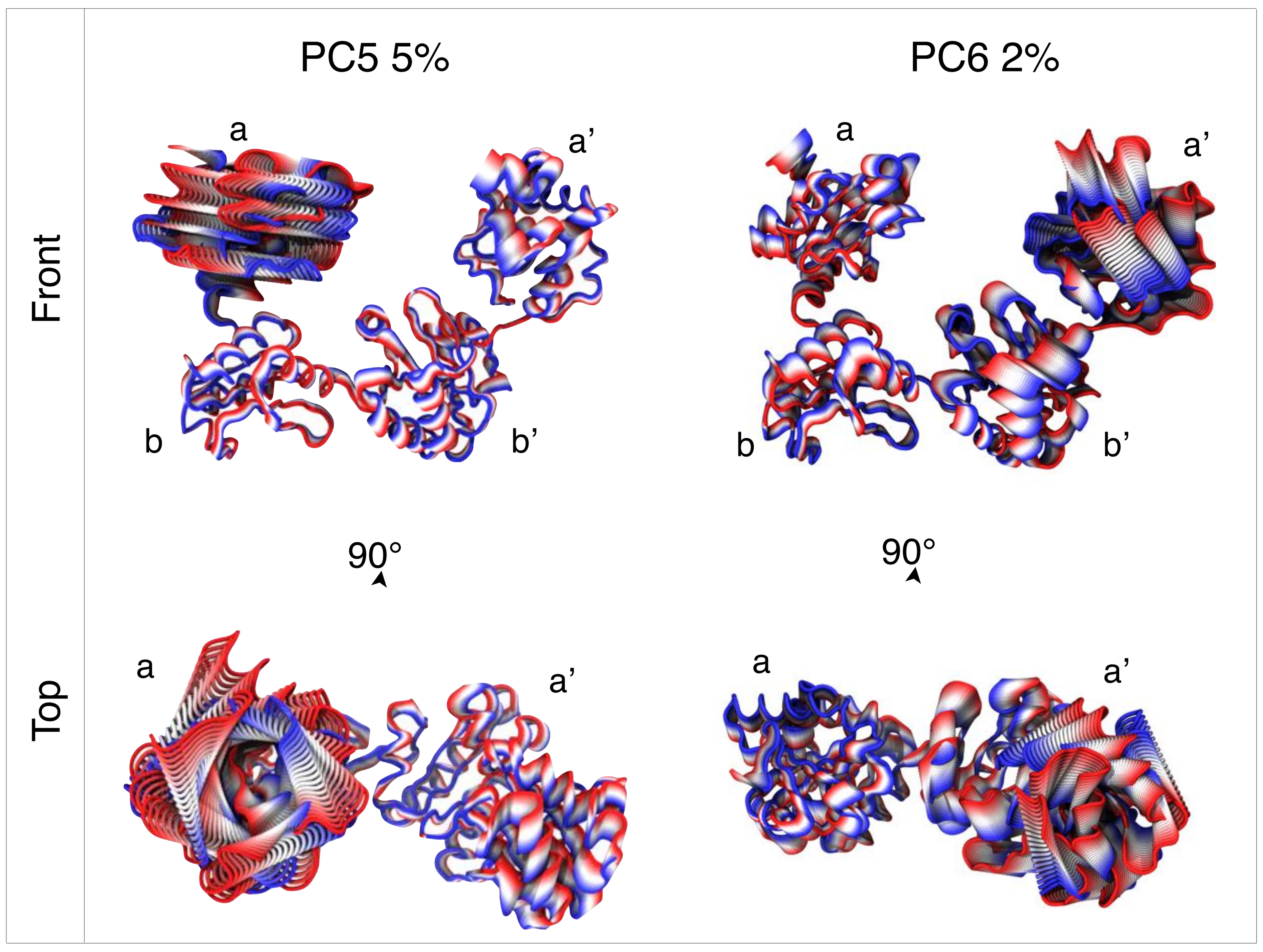

Figure S4 hPDI domain motion modes (PC5 & PC6).Total coverage of variances are represented as % for each PC.

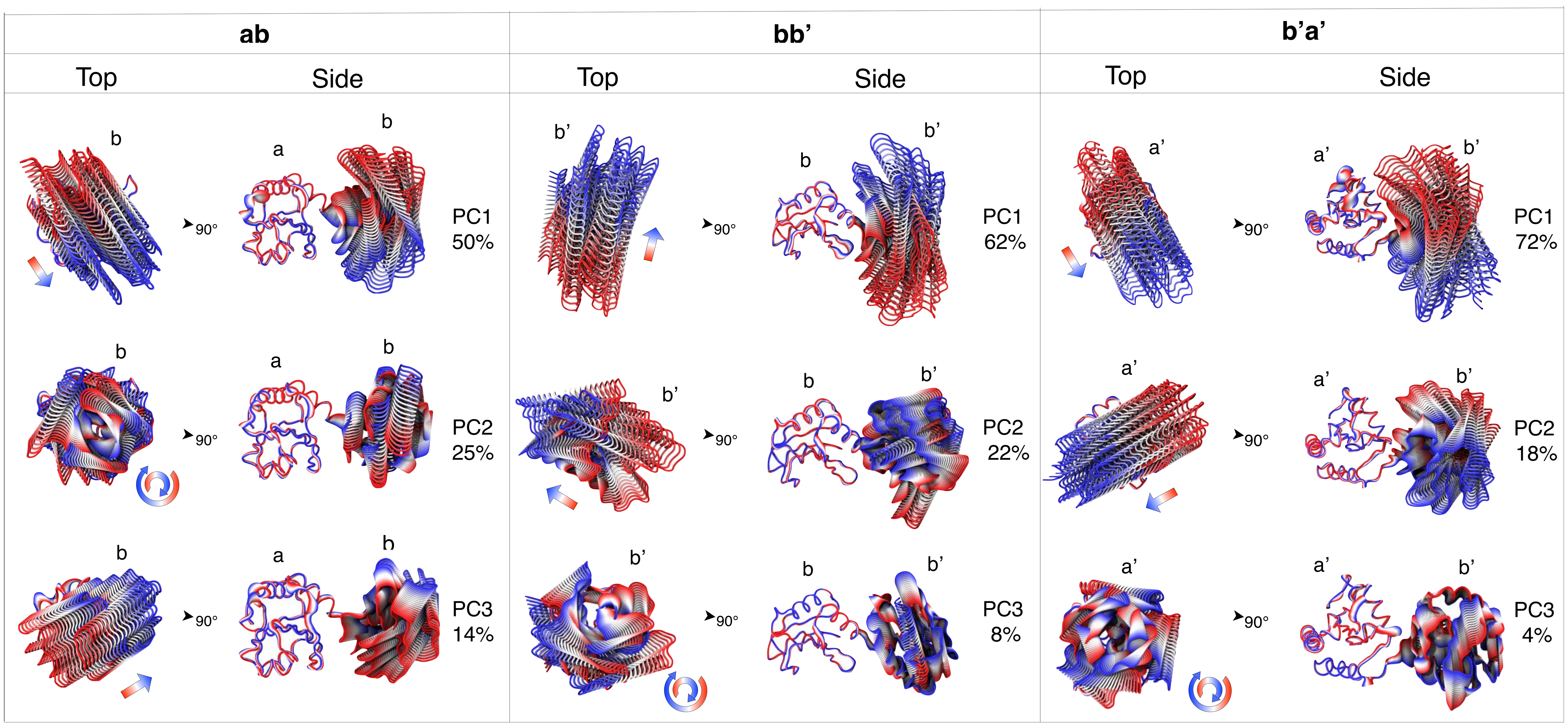

Figure S5 Two domain principal motions of hPDI. Total coverage of variances are represented as % for each PC.

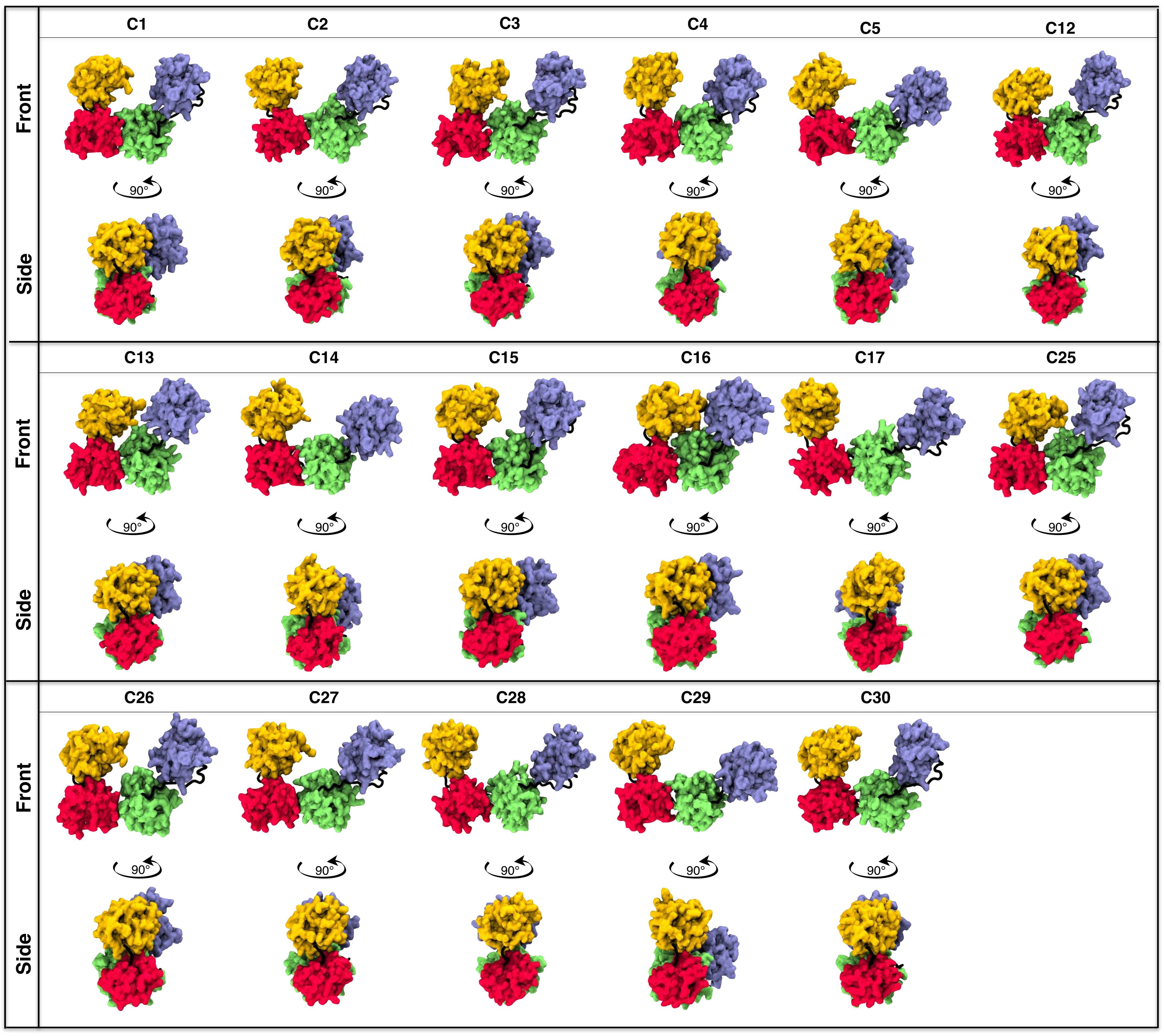

Figure S6 Representative conformations of FEL in oxidized hPDI.

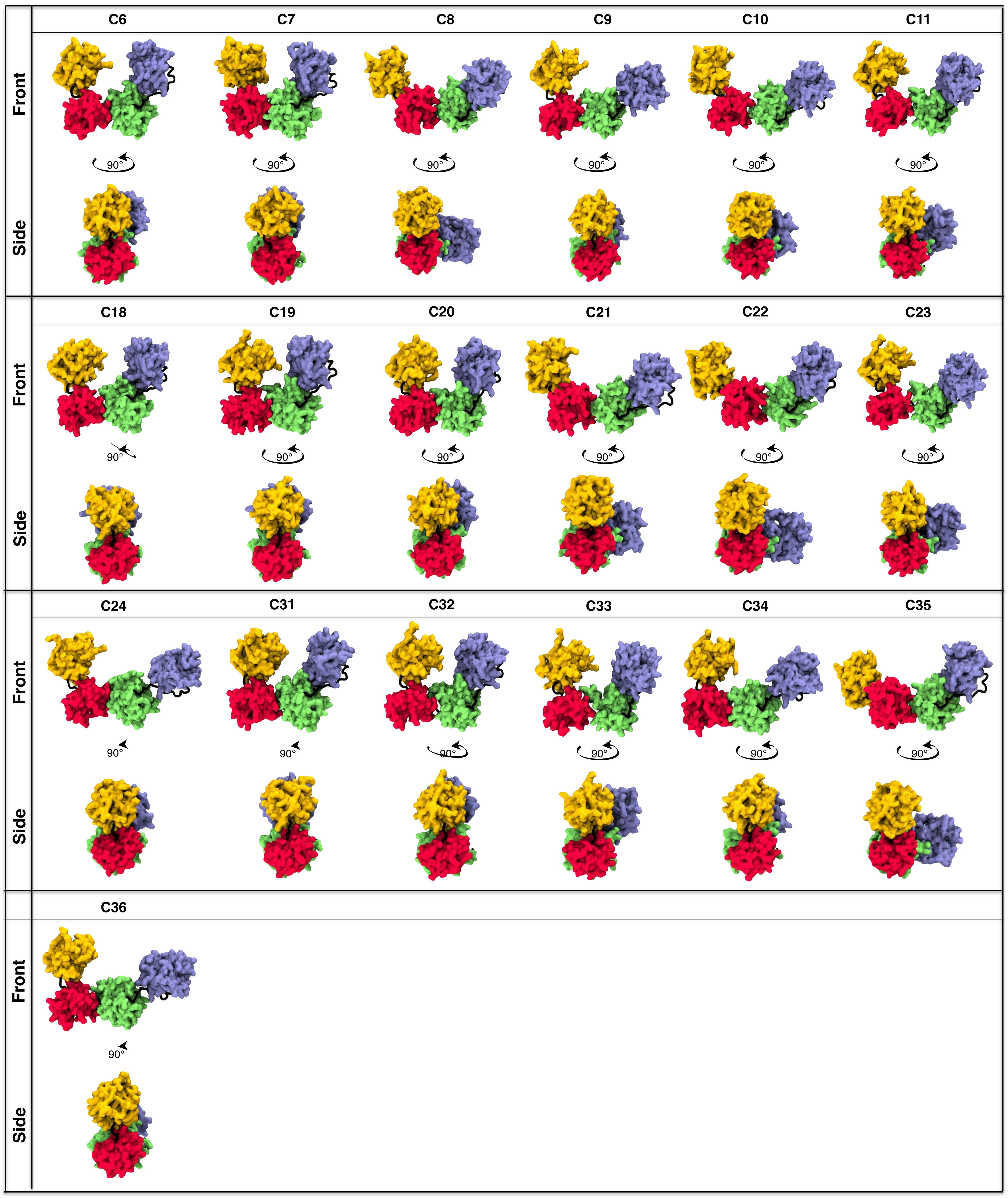

Figure S7 Representative conformations of FEL in reduced hPDI.

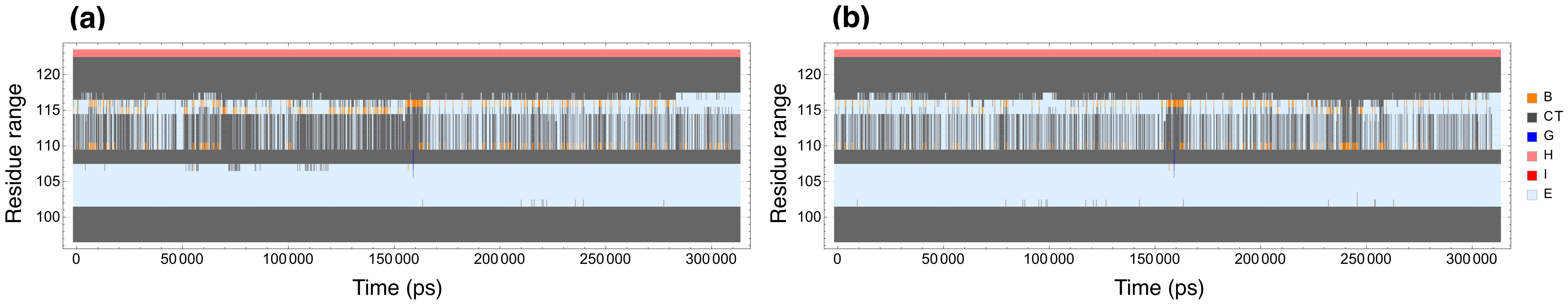

Figure S8 Oscillation frequency of D secondary structure in **(a)** oxidized and **(b)** reduced conformations of hPDI. B: Isolated bridge; C: Coli; G: 3-10 helix; H: Alpha helix; I: Pi helix; E: Extended conformation; CT: Coil and turn.

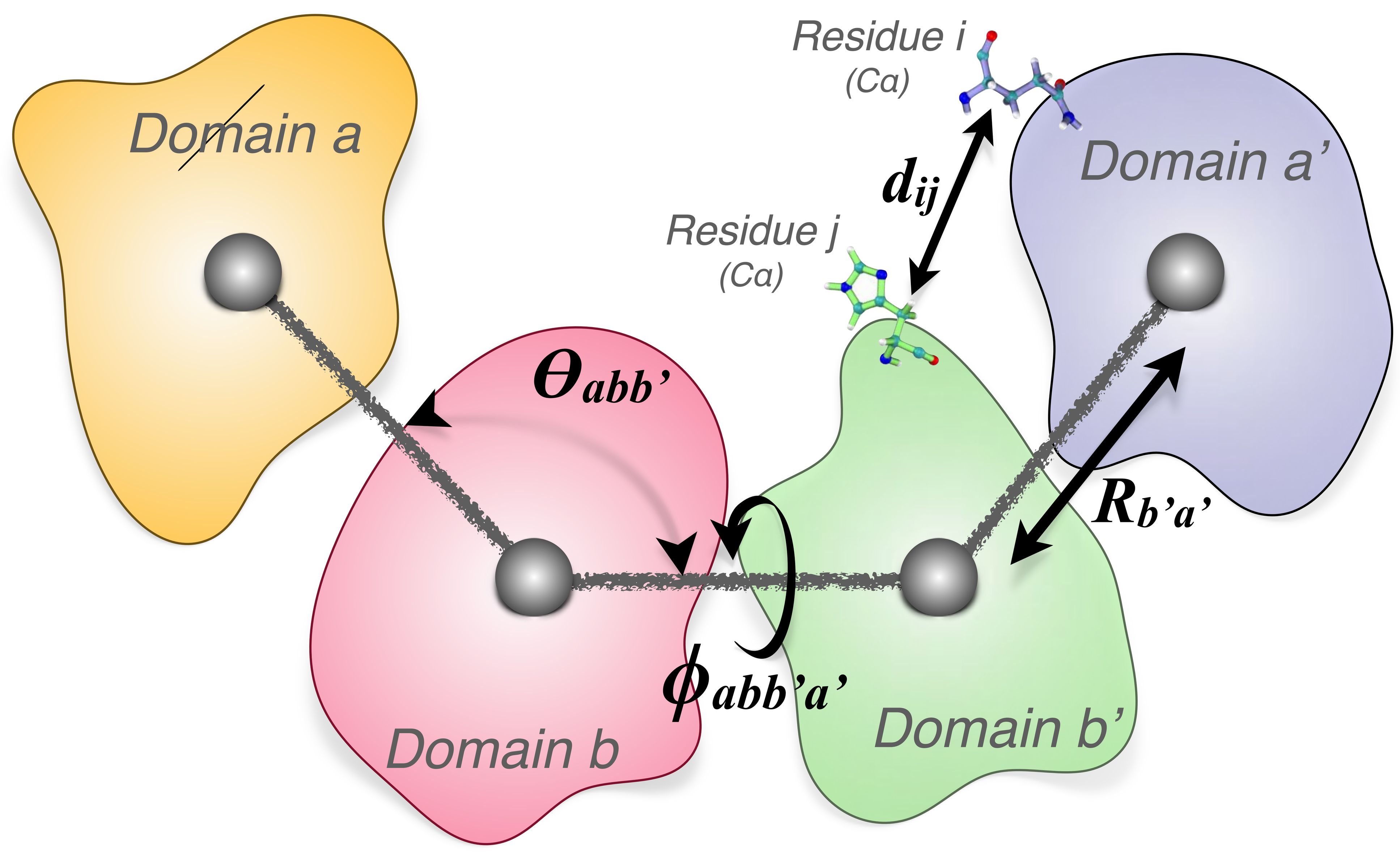

Figure S9 Schematic view of domain- and residue- level structural features.

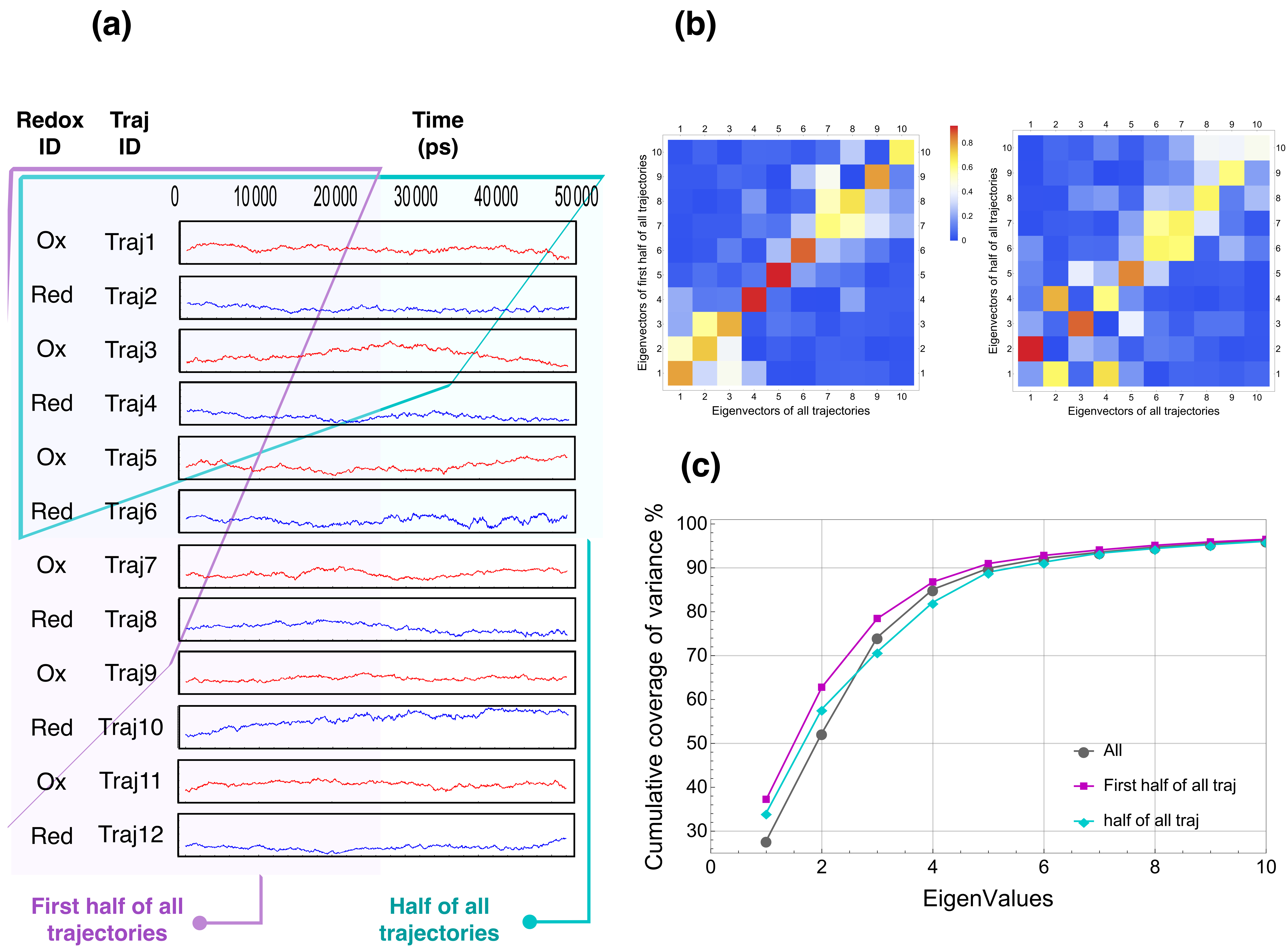

Figure S10 The overlap between whole data and its different halves **(a);** The overlap between eigenvectors of all twelve trajectories versus to the first half of all trajectories (panel left **b**) and also versus to the half of all trajectories (panel right **b**) by evaluation of their scalar product; Values are represented between the range of 0 to 1 with lower and higher overlaps, respectively. (**c**) The cumulative contribution of principal components in total variance in all twelve trajectories, the first half of all trajectories and also the half of all trajectories.

SUPPLEMENTARY TABLES

Table S1 Structural features of representative conformers from FEL. R: inter domain distance; θ: angle; Φ: Dihedral torsion.

index	symbol	frame	R_{ab}	$R_{ab'}$	$R_{aa'}$	$R_{bb'}$	$R_{ba'}$	$R_{b'a'}$	$\theta_{abb'}$	$\theta_{bb'a'}$	$\Phi_{abb'a'}$	PC1	PC2	PC3	PC4	PC5
1	C1	21572	31.4	41.2	48.4	32.2	58.1	36.2	80.7	116.2	40.3	-15.3	-20.6	110.6	26.5	-13.9
2	C2	3364	31.5	47.6	56.8	34.2	61	34.3	92.7	125.9	19.4	24.9	-38	-46.3	71.2	-52.8
3	C3	20565	32	37.4	48	32.8	63.8	38.2	70.5	128	28.4	85.3	-68.8	65.5	-5.3	-7
4	C4	5215	34.3	45.3	49.6	33	59.3	34	84.5	124.4	10	77.8	-7.8	-25.2	45.3	12.8
5	C5	4290	31.7	47.2	63.9	33.2	63.8	35.2	93.3	137.5	36.5	-18	-114	-66.4	-1.8	32.3
6	C6	10534	32.5	46.6	48.7	31.3	54	32.7	93.7	115	21.8	-62.8	86.8	18.6	20.4	14.9
7	C7	25295	32.4	47.7	48.1	30.9	52	31.2	97.7	113.7	6	1.6	92.3	-29	87.2	-72.3
8	C8	28306	33.4	54.8	72.7	32.5	55.4	32	112.5	118.6	85.7	-252.4	-140.4	31	120.9	8.4
9	C9	14656	32.7	45.6	62.9	31.4	62.8	34.5	90.7	145	31.4	-32.5	-103.3	-78.2	-70.7	19.2
10	C10	27825	32.9	53.6	72.9	32.5	59.9	32.2	110.2	135.9	63.2	-225.2	-93.7	-49.3	15.6	2.1
11	C11	26766	31.7	47.8	57.9	33.8	56.2	32.3	93.8	116.5	56.6	-184.9	3.9	96.4	-10.9	26.4
12	C12	7360	31	40.3	49.3	32.3	62.6	38.4	79	124.1	20.9	79.4	-36	19.9	11.1	45.2
13	C13	19248	31.3	37.7	44.6	31.8	59.7	37.2	73.4	119.7	31.4	86.7	-58.6	97.8	48.1	5.5
14	C14	4485	32	44.3	59.1	32.6	63.3	34.9	86.6	139.3	25.5	29.1	-98.8	-55.3	-25.3	32.2
15	C15	21129	31.7	40.7	47.9	32	58.3	36.2	79.5	117.5	39.4	-21	-9.3	103.9	-8.1	-12.4
16	C16	17722	32.5	32.9	37.7	31.9	57.6	34.7	61.4	119.9	33.4	46.4	12.1	146.8	-70.5	-22.3
17	C17	2567	32.8	49.2	69.3	32.7	67.5	37.9	97.3	145.9	-6.2	30.9	-32.1	-197.9	-54.1	-73.5
18	C18	9870	33.1	46.3	48.2	32	55.2	32.1	90.5	119.1	3.5	3.6	103.8	-44.5	-0.5	80.4
19	C19	28935	32.7	42.6	40.9	31.9	53.3	31.5	82.5	114.4	6.3	-0.7	131.5	19.5	-29.3	40.9
20	C20	23659	31.8	44	44.2	32.6	53.2	31.6	86.3	112	23.5	-75.6	103.7	58.6	-33.9	7.7
21	C21	27792	32.2	53.2	72	32.2	58.1	31.6	111.3	131.2	70.7	-226.1	-107.9	-31.4	56.1	8.9
22	C22	28374	33.7	56.6	73	32.1	54.5	32.1	119	116.6	80.1	-255.7	-125.7	13.6	153.1	31.2
23	C23	26653	31.8	46.4	59.9	33.8	59	32.8	90.1	125.1	57.5	-172.9	-30.3	74.3	-54.6	20.3
24	C24	15549	32.1	49.9	71.1	31.6	65.5	36.8	103.2	146.8	20.1	13.2	-139.3	-165.8	12.5	32.3
25	C25	18641	31.3	37.1	43.5	32	59.6	36.3	71.8	121.5	26.7	84.2	-28.2	88.3	21.5	3.3
26	C26	22495	30.5	41	50.1	33.3	60.3	34.1	79.9	127.1	24.5	26.7	-20.7	17.9	-23.3	6.4
27	C27	1349	31.5	41.9	55.5	33.6	64.8	36.1	80.2	136.8	16.4	38.8	-30.3	-55.9	-82.6	-47.6
28	C28	2581	34	49.8	67.6	32.2	66.1	37.5	97.3	143	-9.1	59.7	-11.5	-195.9	-30.9	-53
29	C29	3915	32.9	45.4	65	32.7	64.3	34.8	87.6	145.1	51	-49.3	-156.9	-43.7	-64.7	11.5
30	C30	6592	31	43.8	51.7	33.3	60.1	34	86	126.9	5.5	8.8	63.4	-52.5	-44.5	18.8
31	C31	8816	33.1	43.2	45.2	32.2	56.7	33	82.8	121	-1.9	52.9	89.3	-34.2	-28.8	75.4
32	C32	29128	32.3	42.1	42.7	32	55.1	32.5	81.8	117.3	9.7	-19.3	112.1	25.7	-67	0.9
33	C33	24421	32.1	44.5	48.6	32.3	54.7	32.9	87.2	113.9	38	-105.4	60.4	98.4	-32.7	13.8
34	C34	14370	33.2	43.6	58.6	32.4	64.1	35.6	83.2	140.8	27.8	14.8	-100.3	-39.9	-71.9	-4.1
35	C35	28171	32.8	54.7	73.3	32.1	55.7	31.3	114.8	123.1	84	-255.2	-139.8	6.9	109.9	3.5
36	C36	15299	33.2	46.6	66.3	32.3	66.5	36.5	90.7	150.5	6.5	39.9	-109.8	-154.9	-78.3	8.9

Table S2 Overview of the experimentally point mutations in hPDI compared to the DRIN results (highlighted in red columns).

Reported point mutations	Domain localization	Functional consequences on hPDI	DRIN interaction result with interacting partner(s)	Domain localization of interacting partner(s)	Interaction type	References
**R97E**	a	Decrease reductase activity (～31.8%)	Ox-dominated interactions with E321 and E322	b'	Hydrogen bonds & salt bridges	13
**D180R/D181R**	b	Decrease reductase activity (～73.3%)	Ox-dominated interactions with K254 and K230	b and b'	Salt bridge	
**K326E**	b'	Decrease reductase activity (～44%)	Red-dominated interaction with E431	b' and a'	Hydrogen bonds	
**E431K**	a'	Decrease reductase activity (～46.4%)	Red-dominated interaction with K436, K326 and T325	b' and a'	Salt bridges	
**P235G**	b—b'	Decrease reductase activity (～38.7%)	Red-dominated interactions with Q243 and D210	b and b'	Van der waals	
**R300A**	b'	Similar chaperone activity in the oxidized form but higher activity in the presence of DTT	Red-dominated interactions with W396, P395 and E242	a' and b'	Hydrogen bond & Van der Waals	10
**W396A**	a'		Red-dominated interactions with R300, S427 and F304 Ox-dominated interaction with K401	a' and b'	Hydrogen bond, Van der Waals, pi-pi stacking	
**K326E**	b'		Red-dominated interactions with E431	a'	Hydrogen bond	
**E431K**	a'		Red-dominated interactions with K436, T325 and K326	a' and b'	Hydrogen bond & salt bridges	
